# Interindividual variability in transgene mRNA and protein production following adeno-associated virus gene therapy for hemophilia A

**DOI:** 10.1038/s41591-022-01751-0

**Published:** 2022-04-11

**Authors:** Sylvia Fong, Bridget Yates, Choong-Ryoul Sihn, Aras N. Mattis, Nina Mitchell, Su Liu, Chris B. Russell, Benjamin Kim, Adebayo Lawal, Savita Rangarajan, Will Lester, Stuart Bunting, Glenn F. Pierce, K. John Pasi, Wing Yen Wong

**Affiliations:** 1 BioMarin Pharmaceutical, Novato, CA USA; 2 Department of Pathology, University of California, San Francisco, San Francisco, CA USA; 3 Liver Center, University of California, San Francisco, San Francisco, CA USA; 4 University Hospital Southampton, Southampton, UK; 5 University Hospitals Birmingham, Birmingham, UK; 6 Consultant, La Jolla, CA USA; 7 Barts and the London School of Medicine and Dentistry, London, UK

**Keywords:** Phase I trials, Gene therapy, Phase II trials, Haematological diseases

## Abstract

Factor VIII gene transfer with a single intravenous infusion of valoctocogene roxaparvovec (AAV5-hFVIII-SQ) has demonstrated clinical benefits lasting 5 years to date in people with severe hemophilia A. Molecular mechanisms underlying sustained AAV5-hFVIII-SQ-derived FVIII expression have not been studied in humans. In a substudy of the phase 1/2 clinical trial (NCT02576795), liver biopsy samples were collected 2.6–4.1 years after gene transfer from five participants. Primary objectives were to examine effects on liver histopathology, determine the transduction pattern and percentage of hepatocytes transduced with AAV5-hFVIII-SQ genomes, characterize and quantify episomal forms of vector DNA and quantify transgene expression (hFVIII-SQ RNA and hFVIII-SQ protein). Histopathology revealed no dysplasia, architectural distortion, fibrosis or chronic inflammation, and no endoplasmic reticulum stress was detected in hepatocytes expressing hFVIII-SQ protein. Hepatocytes stained positive for vector genomes, showing a trend for more cells transduced with higher doses. Molecular analysis demonstrated the presence of full-length, inverted terminal repeat-fused, circular episomal genomes, which are associated with long-term expression. Interindividual differences in transgene expression were noted despite similar successful transduction, possibly influenced by host-mediated post-transduction mechanisms of vector transcription, hFVIII-SQ protein translation and secretion. Overall, these results demonstrate persistent episomal vector structures following AAV5-hFVIII-SQ administration and begin to elucidate potential mechanisms mediating interindividual variability.

## Abstract (web summary)

The analysis of liver biopsy samples after AAV gene therapy for hemophilia A reveals normal histology and long-term persistence of the episomal vector, and identifies potential factors contributing to interindividual variability of transgene expression.

## Main

Hemophilia A is an X-linked bleeding disorder caused by deficiency in factor VIII (FVIII) coagulation protein activity.^1^ People with hemophilia A are susceptible to spontaneous and trauma-induced bleeding in soft tissues and joints, resulting in painful, disabling arthropathy, impaired quality of life, possible life-threatening complications such as intracranial hemorrhage, and early death.^1,2^

Hemophilia A is currently managed with chronic administration of exogenous FVIII, either prophylactically or in response to bleeding events, or prophylactic emicizumab, a bispecific antibody that mimics some FVIII functions^1^. Gene therapy with valoctocogene roxaparvovec, a B-domain-deleted human *F8* gene (hFVIII-SQ) with a hybrid liver-selective promoter (HLP) packaged in an adeno-associated virus serotype 5 (AAV5) vector, is being developed for long-term management of severe hemophilia A (FVIII activity < 1 IU dl^−1^).^3–5^ A single intravenous infusion of valoctocogene roxaparvovec (AAV5-hFVIII-SQ) given at a dose of 6 × 10^13^ vector genomes (vg) per kilogram body weight or 4 × 10^13^ vg per kilogram body weight in 13 adults with severe hemophilia A produced clinically relevant FVIII levels and reductions in bleeding and exogenous FVIII usage^4^, with effects lasting for at least 5 or 4 years of follow-up, respectively.^5,6^ The most common adverse event was transient, asymptomatic alanine aminotransferase (ALT) increases that resolved without clinical sequelae.^4,5^ While multiyear expression of FVIII following a single infusion represents a substantial clinical leap forward, there are gaps in our understanding of the biological mechanisms that enable such expression. The molecular fate of the vector genome after administration of AAV-based gene therapy in patients with hemophilia A and host-mediated mechanisms behind interindividual variability in resulting circulating transgene product levels remain to be elucidated.

Multiple, complex processes are involved in achieving successful and durable transgene expression. Following vector infusion, transduction occurs via receptor-mediated uptake into the target cells, intracellular trafficking of the capsids, uptake of vector genomes into the nucleus and processing of vector genomes into stable, full-length forms that can give rise to transgene transcription (Supplementary Fig. 1)^7^. Development of AAV gene transfer for hemophilia A has been challenging because of the size of the *F8* gene, having a coding region (~7.0 kb) larger than the packaging capacity of AAV (4.7 kb^7^). The single-stranded (ss), codon-optimized, B-domain-deleted FVIII genome (hFVIII-SQ) in valoctocogene roxaparvovec has been developed to address this limitation, but at >4.9 kb is still over the normal packaging limit of AAV^3^; therefore, the ssDNA packaged into the capsid may be incomplete, and assembly and repair of two complementary ssDNA genomes into a full-length structure may be required. Studies in lung, liver and muscle transduced by AAV gene therapy with normal-sized vectors (<4.7 kb^7^) in mice and nonhuman primates (NHPs) have shown that circularized monomeric and concatemeric episomes are the major DNA species associated with long-term, persistent expression of the gene product in the target cell^8–16^. More recently, we have similarly demonstrated that long-term FVIII expression from AAV5-hFVIII-SQ transduction in mice and NHPs is associated with formation of full-length circular episomes in the liver^17^. Whether this is the case in human liver has not been previously determined.

In the present study, we used liver biopsy samples from men with severe hemophilia A who participated in a clinical trial of gene transfer with valoctocogene roxaparvovec, to characterize vector genome distribution, episomal forms and expression of vector DNA that persists multiple years after gene transfer. These findings provide insights into the complex molecular mechanisms behind successful in vivo transduction of the human liver using an AAV gene therapy platform. We also looked for any safety concerns at the tissue level and explored potential mechanisms mediating interindividual variability. Alongside similar investigations in mice and NHPs, reported separately^17^, the results also further our knowledge of interspecies translatability in gene therapy.

## Results

All 15 men with severe hemophilia A who enrolled in the phase 1/2 clinical trial of AAV5-hFVIII-SQ^4,5^ were invited to take part in this liver biopsy substudy, five of whom consented to participate. The objectives were to examine any effects of the vector on liver histopathology, to determine the pattern and extent of AAV5-hFVIII-SQ transduction and episomal forms of vector DNA in the hepatocytes and to quantify transgene expression by evaluation of hFVIII-SQ RNA and hFVIII-SQ protein levels. Substudy participants had received a single infusion of AAV5-hFVIII-SQ at one of three dose levels: 6 × 10^12^ vg per kg body weight (one participant), 4 × 10^13^ vg per kg body weight (two participants) or 6 × 10^13^ vg per kg body weight (two participants; Table 1)^4,5,18^. Transjugular or ultrasound-guided percutaneous liver biopsy was performed 2.6–4.1 years after infusion by the preferred standard procedures at the local institution.

**Table 1:** Key demographic and baseline clinical characteristics of adult male participants with severe hemophilia A who underwent liver biopsy in the phase 1/2 clinical trial of valoctocogene roxaparvovec (AAV5-hFVIII-SQ)

^a^Participant	Dose of AAV5-hFVIII-SQ, vg per kg body weight	^b^Age at first enrollment, years	Biopsy time point (after first enrollment), weeks (years)	Biopsy date	Route of biopsy	No. of hepatic lobules in biopsy sample	ALT at time of biopsy, U l^−1^	^c^FVIII activity at time of biopsy, IU dl^−1^	
								CS result	OS result
1^d^	6 × 10^12^	25	201 (3.86)	Aug 2019	Transjugular	12	29	BLD	BLD
11	4 × 10^13^	37	140 (2.69)	Aug 2019	Transjugular	23	11	18.6	28.4
15	4 × 10^13^	37	148 (2.85)	Jan 2020	Percutaneous	15	20	BLD	2.1
3	6 × 10^13^	32	214 (4.12)	Jan 2020	Percutaneous	23	12	8.2	14
4	6 × 10^13^	23	213 (4.10)	Mar 2020	Percutaneous	18	11	13.5	23.9

^a^Participants were numbered according to the order in which they were enrolled and dosed in the clinical trial^4,5^.

^b^Age at enrollment into the valoctocogene roxaparvovec phase 1/2 clinical trial (NCT02576795).

^c^FVIII levels were measured using both a one-stage (OS) activated partial thromboplastin time-based clotting assay and a chromogenic substrate (CS) assay^18^.

^d^Participants were admitted to hospital on the morning of the procedure and all except participant 1 were discharged on the following day as planned. Participant 1 experienced abdominal pain during the procedure and had post-procedural bleeding. Computed tomography scan revealed a small hepatic hematoma with no evidence of active bleeding or vascular injury. The participant was treated with FVIII replacement and discharged 2 d after the biopsy procedure due to clinical improvement. The participant was readmitted 1 week after discharge with abdominal pain, vomiting and orthostatic hypotension; repeat computed tomography revealed old blood within the liver capsule and a liver laceration but no active bleeding. Although there were complications involving a fever, the participant was managed successfully with supportive treatment and finally discharged 3 weeks after the biopsy procedure.

BLD, below limit of detection.

### Liver histopathology following AAV5-hFVIII-SQ gene transfer

**Fig. 1:**
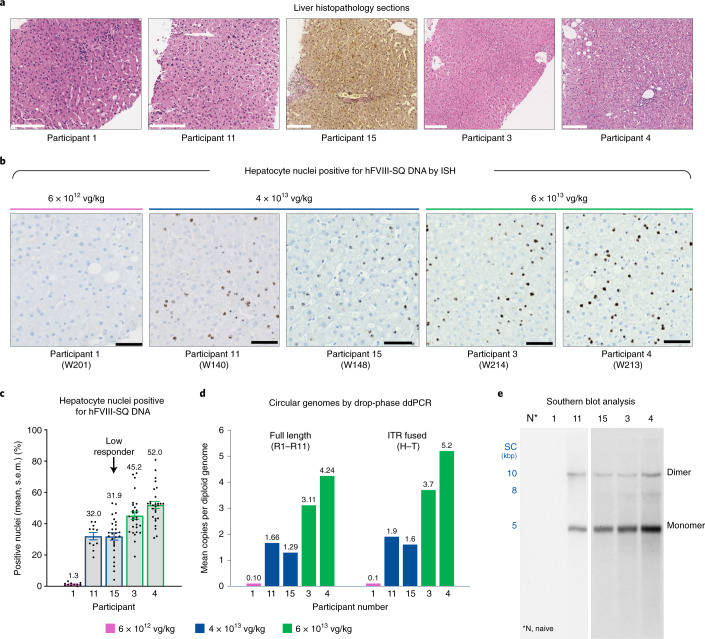
Histopathology and hFVIII-SQ DNA transduction efficiency in liver biopsy samples from five participants 2.6–4.1 years after gene transfer with valoctocogene roxaparvovec. **a**, Representative liver histopathology sections stained with H&E (participants 1, 3, 4 and 11) or hematoxylin and Van Gieson (participant 15); histologic sections per participant were reviewed by the local pathologist and central pathologist, with consistent results: three biopsy levels (participant 1), two levels (participants 11 and 15), one level (participant 3) and four levels (participant 4). Images were captured in QuPath v0.2.3 using the export snapshot feature; no subsequent processing or image enhancement was performed. Scale bars, 180 µm (participant 1); 150 µm (participants 11, 15, 3 and 4) **b**, Representative liver biopsy sections from each participant showing hFVIII-SQ DNA (brown foci) by ISH. Images were captured at 1,600 × 1,200 pixels and output at 300 pixels per inch (ppi). Each focus (brown dot) represents at least one vector DNA molecule; it is possible to have multiple copies of vector genome within a single focus. Scale bars, 50 µm. **c**, Percentage of hepatocyte nuclei stained positive for hFVIII-SQ DNA by ISH. Data are means across 11 (participants 1 and 11) or 27–28 (participants 3, 4 and 15) images per biopsy section, spanning ≥50% of the tissue area (biopsy tissue area was larger for participants 3, 4 and 15). Error bars represent the s.e.m., dots represent quantification of each individual image, and data labels show mean values. **d**, Circular genomes (full length or H–T ITR fused) detected in liver biopsy samples via drop-phase ddPCR following DNA sample treatment with PS-DNase and KpnI (with PS-DNase, all linear forms of DNA are hydrolyzed, and only circular forms of DNA remain; KpnI treatment separates out vector genome units within the concatemeric forms, enabling quantification of genome units within concatemeric vector genomes). **e**, Qualitative Southern blot analysis of circular episomes after PS-DNase treatment of DNA from liver biopsy samples. Biopsy samples from control, participant 1 and participant 11, and for participants 3, 4 and 15 were processed at separate times; results are presented on separate blots from two independent experiments, and are not intended to present a quantitative comparison. kbp, kilobase pairs; SC, supercoiled markers; W, week. Source data

**Extended Data Fig. 1:**
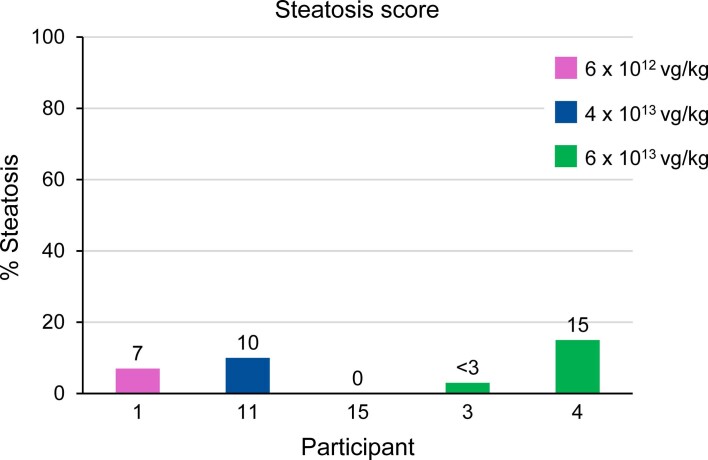
Histopathological assessment of liver biopsy samples from five participants 2.6–4.1 years after gene transfer with valoctocogene roxaparvovec. Steatosis scored as percentage of lipid-containing hepatocytes.

Liver biopsy samples were evaluated for any adverse histopathological findings by pathologists at the local sites and centrally by an independent expert liver pathologist. No clinically relevant inflammation was observed (Extended Data Table 1): biopsy samples showed mostly sinusoidal infiltrates, common in liver biopsy samples without definitive chronic disease^19^. There was no notable fibrosis and no evidence of dysplasia, necrosis or architectural distortion (Fig. 1a). Mild steatosis was observed in four of the five participants (Extended Data Fig. 1). A single sample from participant 11 (4 × 10^13^ vg per kg body weight) showed rare occurrence of macrophages, suggesting a possible acute resolving event in the previous month, but no associated fibrosis, necrosis, apoptosis, neoplastic changes or findings of chronic liver disease.

### AAV5-hFVIII-SQ transduced hepatocytes throughout biopsy samples

**Extended Data Fig. 2:**
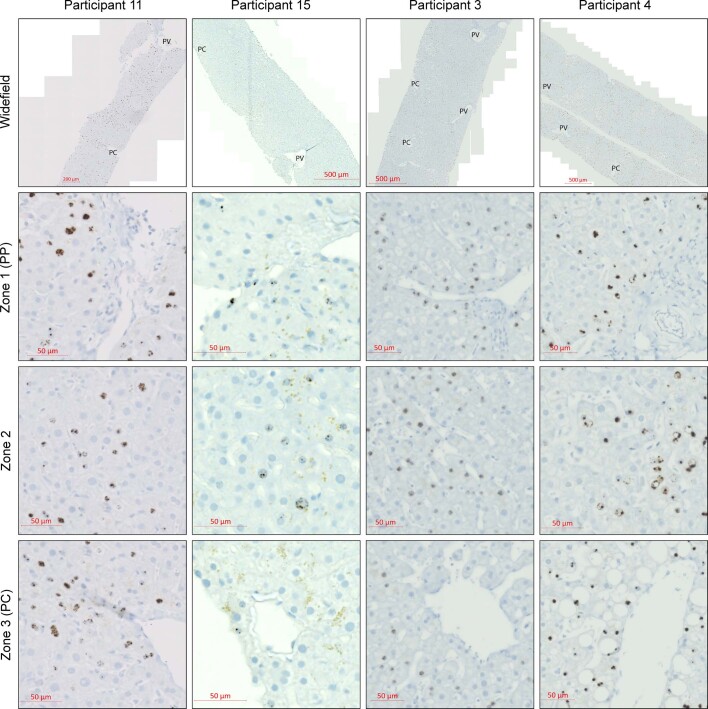
Valoctocogene roxaparvovec transduction patterns in hepatocytes amongst the four participants with > 2% hepatocytes stained positive. In situ hybridization analysis to detect vector genomes, showing unbiased distribution of hFVIII-SQ DNA (brown foci) in liver biopsy samples of Participants 11, 15, 3, and 4; Participant 1 has less than 2% of hepatocytes stained positive for vector DNA and did not have any identifiable veins (PC or PV) as the biopsy was broken into many pieces, thus not shown. Each focus (brown dot) represents at least one vector DNA molecule; it is possible to have multiple copies of vector genome within a single focus. Zoom-out (widefield) images allow comparison of liver sections proximal and distal to central veins. Zoom-in images separated according to liver zones – zone 1 being peri-portal vein (PP), zone 3 being peri-central vein (PC), and zone 2 being in between zone 1 and 3. Images were captured at 1600 × 1200 pixels and output at 300 ppi. PV, portal vein.

To evaluate the pattern and extent of AAV5-hFVIII-SQ vector transduction following gene transfer, hepatocellular distribution of hFVIII-SQ DNA in formalin-fixed, paraffin-embedded (FFPE) liver biopsy samples was determined by in situ hybridization (ISH). Although only a single biopsy was performed on each participant, each sample comprised ≥10 hepatic lobules (Table 1), allowing a limited evaluation of vector distribution. An increasing trend in the percentage of hepatocytes that were stained positive for vector genomes was observed across the three dose levels: 1.3% (6 × 10^12^ vg per kg body weight), 32% (4 × 10^13^ vg per kg body weight) and 45–52% (6 × 10^13^ vg per kg body weight), with most positive nuclei containing multiple foci (Fig. 1b,c). Interestingly, despite the two participants who received the 4 × 10^13^ vg per kg body weight dose having similar levels of vector genome-positive cells, circulating FVIII activity levels differed more than tenfold between them (Table 1). Positive staining for hFVIII-SQ DNA was detected in all zones within the hepatic lobule; regarding the four participants with >2% of hepatocytes that stained positive for vector DNA, there was no bias of genome distribution in cells across zones in 79 hepatic lobules examined (Extended Data Fig. 2). We hypothesized that the distribution of AAV5-hFVIII-SQ in human liver would coincide with expression of AAV5 uptake receptors, as described in animal models (Supplementary Fig. 2a). In normal human liver, AAV pan-receptor (AAVR) and AAV5 co-receptor, PDGFRA, are evenly distributed across all three zones within hepatic lobules (Supplementary Fig. 2b), supporting a non-biased pattern of transduction consistent with the unbiased distribution of vector genomes detected in the human biopsy samples in the present study.

### Presence of full-length hFVIII-SQ vector DNA in circular episomal forms

Following transduction of hepatocytes by the over-sized AAV vector, assembly and repair of functional full-length vector genomes is required. The viral inverted terminal repeats (ITRs) at each end of the vector genome drive recombination through ITR fusion to form stable circularized episomal genomes that can persist in the nucleus (Supplementary Figs. 1 and 3)^7,20^. We characterized the different molecular forms of hFVIII-SQ vector genomes in the cells by quantitative, drop-phase droplet digital PCR (ddPCR; Supplementary Fig. 4a) and by qualitative Southern blotting analyses performed on DNA isolated from biopsy samples that were treated with various DNA digestion enzymes, and using custom-generated primers/probe sets (Supplementary Fig. 4b and Supplementary Table 1).

**Extended Data Fig. 3:**
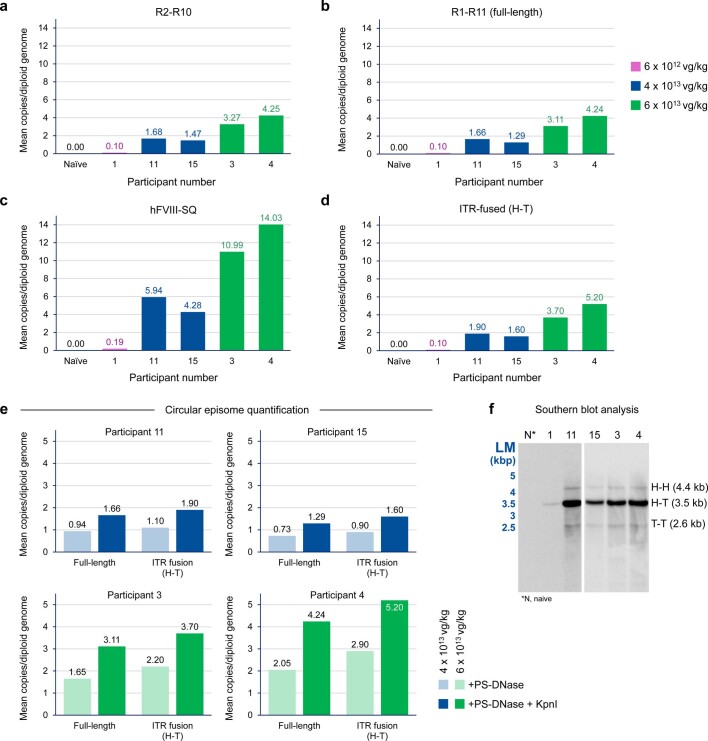
Quantification of circular vector genomes [DNA] and assessment of multimeric circular episomal forms in liver samples from patients. Quantification of circular vector genomes [DNA] as measured by ddPCR utilizing primers and probes to detect specific regions of the vector genome: **a)** R2-R10 linked amplicons. **b)** R1-R11 linked amplicons (full-length). **c)** SQ amplicon (representing overall vector genome count). **d)** ITR-fused amplicons; ITR fusion assay measured 5′ to 3′ ITR recombination (that is head-to-tail, H-T). **e)** Circular multimeric episome quantification following PS-DNase (eliminates all linear forms of DNA) or PS-DNase/KpnI digestion (to separate individual vector genome units within concatemeric circular forms). ITR fusion assay measured 5′ to 3′ ITR recombination (that is head-to-tail, H-T). **f)** Qualitative Southern blot assessment of vector genome configuration in circular forms obtained after DNA treatment with PS-DNase and KpnI. DNA fragments corresponding to 3.5 kb, or 4.4 and 2.6 kb could be detected for H-T or H-H/T-T concatemer configurations, respectively. Biopsies from Control, Participant 1, and Participant 11, and for Participants 3, 4, and 15 were processed at separate times; results are presented on separate blots from two independent experiments, and are not intended to present a quantitative comparison. ddPCR, droplet digital polymerase chain reaction; H, head (5′ end); H-H, head-to-head orientation; H-T, head-to-tail orientation; ITR, inverted terminal repeat; kb, kilobase pairs; LM, linear markers; PS-DNase, Plasmid Safe™ ATP-Dependent DNAase; T, tail (3′ end); vg, vector genome. ddPCR was performed after DNA digestion with PS-DNase and KpnI (to eliminate linear forms and to separate individual vector genome units within concatemeric circular forms). Source data

Full-length circular vector genomes were detected in participants’ liver biopsy samples 2.6–4.1 years after a single AAV5-hFVIII-SQ gene transfer, at levels showing a trend for dose dependence (Fig. 1d). Consistent with the ISH analysis of hepatocyte transduction, the two participants who received the 4 × 10^13^ vg per kg body weight dose had similar levels of full-length circular genomes. ITR fusion analysis revealed that levels of head-to-tail (H–T) ITR fusions were similar to levels of full-length genomes (Fig. 1d), suggesting that most ITR-fused genomes are full length. Levels of R2–R10-linked vector genomes (to quantify ITR-deleted genomes; Supplementary Fig. 4b) were similar to those of full-length (R1–R11) genomes (Extended Data Fig. 3a,b), suggesting that minimal deletion of the D-loop region of ITRs occurred. Of all structures surviving Plasmid-Safe ATP-dependent DNase (PS-DNase) treatment, which degraded >99% of linear DNA (Supplementary Table 2), 30–40% of those containing the central portion of the transgene (SQ amplicon) were full length or ITR fused (Extended Data Fig. 3b–d). This indicates that some circular episomes were incomplete, missing either the 5′ or the 3′ ITR, and suggests that adjacent promoter or polyA regions (Supplementary Fig. 4) might also be truncated, resulting in vector genomes that are unable to produce functional FVIII RNA or protein.

Circular episomes can be monomeric or include several genome copies linked end to end (concatemers).^20^ In drop-phase ddPCR analysis, whole concatemers each make one count, thereby undercounting the total number of vector genome units. DNA samples treated with PS-DNase followed by KpnI restriction enzyme digest to separate individual vector genome units showed higher levels of genomes compared with samples treated with PS-DNase alone, confirming the presence of concatemeric vector genomes (Supplementary Fig 4c and Extended Data Fig. 3e). Consistent with ddPCR results, Southern blotting, utilizing two probes annealing to the 5′ (H) and 3′ (T) ends of the vector genome (Supplementary Fig 5 and Supplementary Table 3), showed that circular episomes were present in monomeric (major species) and concatemeric forms at sizes corresponding to full-length genomes (Fig. 1e and Supplementary Fig. 5). Circular vector genomes were primarily in H–T configuration (Extended Data Fig. 3f), as reported when AAV5-hFVIII-SQ was administered to mice and NHPs^17^.

### Measurement of hFVIII-SQ RNA expression

**Fig. 2:**
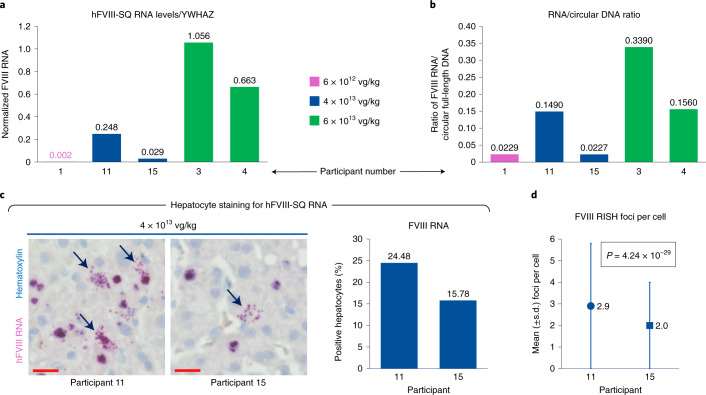
Detection of hFVIII-SQ transcript in adult liver biopsy samples. **a**, Levels of hFVIII-SQ RNA detected in adult liver samples normalized to endogenous reference RNA (YWHAZ). **b**, Ratio of hFVIII-SQ RNA to circular full-length DNA (R1–R11-linked DNA, PS-DNase + KpnI). **c**, Representative images of liver biopsy sections from participants who received a dose of 4 × 10^3^ vg per kg body weight (participants 11 and 15; evaluation of one section each), showing hepatocytes that stained positive for hFVIII-SQ RNA by RISH (arrows indicate cytoplasmic staining of AAV5-FVIII-SQ-derived RNA), and the percentage of hepatocytes staining positive for hFVIII-SQ RNA. Images were captured at 1,600 × 1,200 pixels and output at 300 ppi. Scale bars, 20 µm. **d**, hFVIII-SQ RNA staining signal per cell in 4 × 10^3^ vg per kg body weight-dosed participants; *P* = 4.24 × 10^−29^ for participant 11 (*n* = 2,212 cells examined in one slide) versus participant 15 (*n* = 1,397 cells examined in one slide), two-tailed unpaired *t*-test. YWHAZ, tyrosine 3-monooxygenase/tryptophan 5-monooxygenase activation protein zeta. Source data

**Extended Data Fig. 4:**
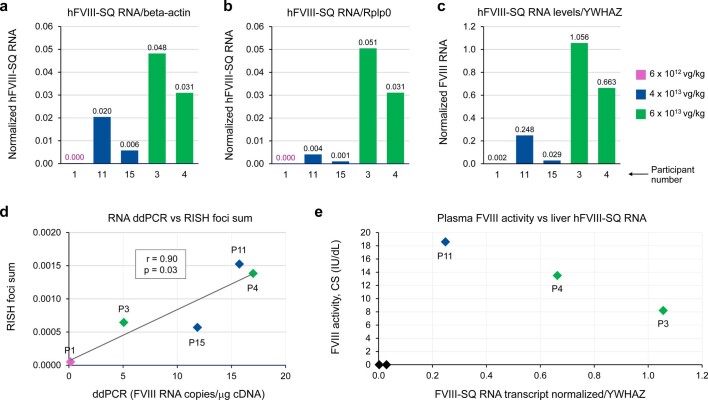
Detection of hFVIII-SQ RNA transcript in patient liver biopsy samples. hFVIII-SQ RNA levels detected in patient liver samples normalized to endogenous reference RNAs **a)** beta-actin, **b)** Rplp0, and **c)** YWHAZ. **d)** Correlation of RNA quantified by ddPCR with total RISH area signal in one tissue section per participant; RISH foci sum normalized to tissue area, μm^2^ (Pearson correlation coefficient, r = 0.90; p = 0.03, two-tailed). **e)** Correlation between plasma FVIII activity and liver hFVIII-SQ RNA; Participants 1 and 15 had no or barely detectable plasma FVIII activity (black diamonds). CS, chromogenic substrate assay; hFVIII-SQ, B domain-deleted human factor VIII; P, participant; RISH, RNA in situ hybridization; Rplp0, ribosomal protein lateral stalk subunit P0; YWHAZ, tyrosine 3-monooxygenase/tryptophan 5-monooxygenase activation protein zeta. Source data

In assessments of hFVIII-SQ vector genome transcriptional efficiency, hFVIII-SQ RNA was detected in all participants. In general, higher levels of hFVIII-SQ RNA were observed at higher doses of AAV5-hFVIII-SQ, regardless of the endogenous housekeeping genes used for normalization (Fig. 2a and Extended Data Fig. 4a–c), resulting in RNA/DNA ratios that followed the same pattern for each participant (Fig. 2b). Notably, however, at the same dose level of AAV5-hFVIII-SQ (4 × 10^13^ vg per kg body weight), participant 15 had approximately tenfold lower hFVIII-SQ RNA levels than participant 11, while transduction of hFVIII-SQ vector genome DNA appeared equally effective, as reported above; the RNA/DNA ratio was therefore correspondingly low in participant 15 (Fig. 2b). The lower RNA level in participant 15 was a result of both a lower percentage of hepatocytes expressing hFVIII-SQ RNA (Fig. 2c) and less hFVIII-SQ RNA produced per cell (Fig. 2d). hFVIII-SQ RNA data quantified by ddPCR correlated with total hFVIII-SQ RNA in situ hybridization (RISH) area signal (Extended Data Fig. 4d). These findings are consistent with a marked difference between the two participants in plasma FVIII activity reported at the time of biopsy (Table 1).

### Potential mechanisms contributing to interindividual variability

**Fig. 3:**
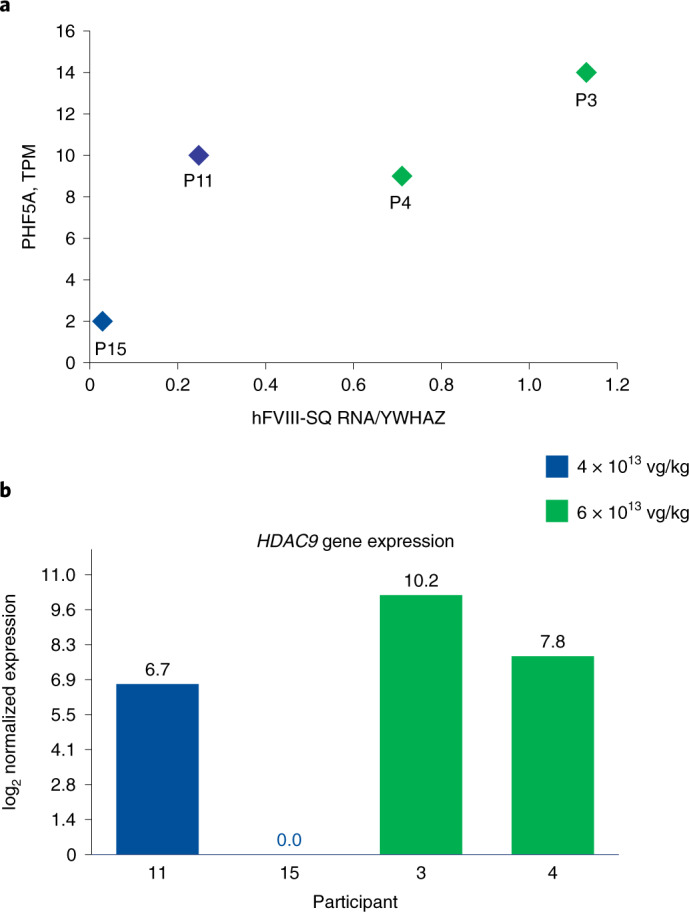
Differential gene expression of selected genes potentially mediating low hFVIII-SQ RNA levels in participant 15. **a**, Relationship between PHF5A expression and normalized hFVIII-SQ RNA levels in liver biopsy samples of participants (P) who received 4 × 10^13^ vg per kg body weight (blue) or 6 × 10^13^ vg per kg body weight (green) valoctocogene roxaparvovec. **b**, *HDAC9* gene expression in participants who received 4 × 10^13^ vg per kg body weight (blue) or 6 × 10^13^ vg per kg body weight (green) valoctocogene roxaparvovec. TPM, transcripts per million. Source data

**Extended Data Fig. 5:**
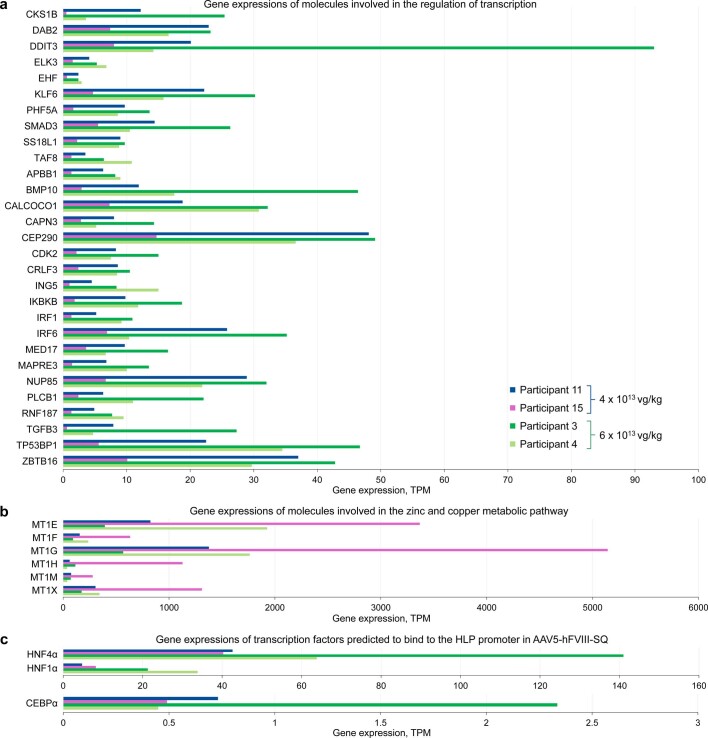
Gene expression of molecules involved in the regulation of hFVIII-SQ RNA expression. **a)** Down-regulation in Participant 15 (low-responder) of molecules involved in positive regulation of transcriptional pathways. **b)** Upregulation in Participant 15 (low-responder) of zinc and copper metabolic pathway negatively correlated with liver hFVIII-SQ RNA levels. **c)** Expression levels of transcription factors predicted to bind to the hybrid liver-specific promoter (HLP) in AAV5-hFVIII-SQ. TPM, transcripts per million. *Gene names and descriptions: a. Genes clustered in the regulation of transcription, DNA templated pathway*. **CKS1B**, CDC28 protein kinase regulatory subunit 1B; **DAB2**, Dab, mitogen-responsive phosphoprotein, homolog 2 (*Drosophila*); **DDIT3**, DNA-damage-inducible transcript 3; **ELK3**, ELK3, ETS-domain protein (SRF accessory protein 2); **EHF**, ets homologous factor; **KLF6**, Kruppel-like factor 6; **PHF5A**, plant homeodomain (PHD) finger protein 5A; **SMAD3**, SMAD family member 3; **SS18L1**, synovial sarcoma translocation gene on chromosome 18-like 1; **TAF8**, TAF8 RNA polymerase II, TATA box binding protein (TBP)-associated factor, 43 kDa; **APBB1**, amyloid beta (A4) precursor protein-binding, family B, member 1 (Fe65); **BMP10**, bone morphogenetic protein 10; **CALCOCO1**, calcium binding and coiled-coil domain 1; **CAPN3**, calpain 3, (p94); **CEP290**, centrosomal protein 290 kDa; **CDK2**, cyclin-dependent kinase 2; **CRLF3**, cytokine receptor-like factor 3; **ING5**, inhibitor of growth family, member 5; **IKBKB**, inhibitor of kappa light polypeptide gene enhancer in B cells, kinase beta; **IRF1**, interferon regulatory factor 1; **IRF6**, interferon regulatory factor 6; **MED17**, mediator complex subunit 17; **MAPRE3**, microtubule-associated protein, RP/EB family, member 3; **NUP85**, nucleoporin 85 kDa; **PLCB1**, phospholipase C, beta 1 (phosphoinositide-specific); **RNF187**, ring finger protein 187; **TGFB3**, transforming growth factor, beta 3; **TP53BP1**, tumor protein p53 binding protein 1; **ZBTB16**, zinc finger and BTB domain containing 16. *b. Genes clustered in the Zinc and Copper Metabolism pathway*. **MT1**, metallothionein 1. *c. Transcription factors predicted to bind to the HLP promoter in AAV5-hFVIII-SQ*. **CEBPa**, CCAAT/enhancer-binding protein alpha; **HNF1a**, hepatocyte nuclear factor 1 alpha; **HNF4a**, hepatocyte nuclear factor 4 alpha. Source data

**Extended Data Fig. 6:**
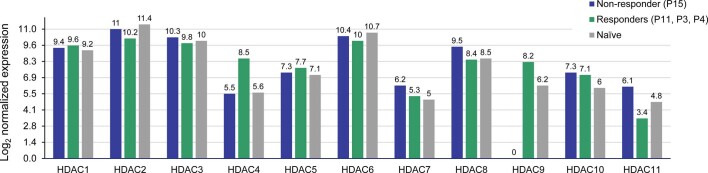
Log2 normalized expression of all *HDAC* genes based on responding vs non-responding status. *HDAC* RNA expression in non-responder Participant 15 (blue), responders (average of Participants 11, 3, and 4; green), and in naïve human liver (grey). HDAC, histone deacetylase. Source data

To investigate molecular mechanisms mediating variability in hFVIII-SQ RNA levels, RNA-sequencing (RNA-seq) analyses were performed in participants 3, 4, 11 and 15. Lists of genes whose expression correlated with levels of hFVIII-SQ RNA (*r* ≥ 0.9) and with differential expression (2.5-fold) between the responders (participants 3, 4 and 11) and nonresponder (participant 15) were subjected to pathway enrichment analysis. Responder versus nonresponder differential expression analysis yielded significant correlation with transcription machinery. Molecules involved in positive regulation of transcriptional pathways were downregulated in participant 15 (Extended Data Fig. 5a). In particular, expression of the *PHF5A* (plant homeodomain finger protein 5A) gene, involved in transcriptional elongation by RNA polymerase II and pre-mRNA splicing, was five- to seven-fold lower in participant 15 compared to participants 3, 4 and 11 (Fig. 3a). Conversely, molecules involved in zinc and copper metabolism were upregulated in participant 15 (Extended Data Fig. 5b); the effect of differential expression in these pathways is currently unclear as the role of metal ions in AAV-mediated gene expression is largely unexplored^21^. We also evaluated expression levels of transcription factors predicted to bind to the HLP promoter in AAV5-hFVIII-SQ, including hepatocyte nuclear factors HNF1A and HNF4A, and CCAAT/enhancer-binding protein alpha (CEBPA); none of these was significantly different between the responder and nonresponder dosed at 4 × 10^13^ vg per kg body weight (Extended Data Fig. 5c). The transcriptional activities mediated by HNF1A, HNF4A and CEBPA have been shown to be regulated by acetylation status.^22–24^ Therefore, one possible explanation is that these transcription factors, although present in normal amounts, have an altered acetylation status in participant 15. Of note, expression of histone deacetylase 9 (HDAC9), involved in deacetylating histones and other proteins^25,26^, was evident in participants 3, 4 and 11 but not detected in participant 15 (Fig. 3b), although other HDACs analyzed showed minimal or no difference in expression among participants (responders or nonresponder) and naïve healthy human liver (Extended Data Fig. 6). Whether the observed variability in hFVIII-SQ RNA levels is related to possible alterations in the acetylation status of the transcription factors or the differential transcription of hFVIII-SQ remains to be determined.

### Measurement of hFVIII-SQ protein expression and endoplasmic reticulum stress

Initial immunohistochemistry (IHC) followed by epifluorescence microscopy to detect hFVIII-SQ protein expression and distribution in hepatocytes was unsuccessful presumably due to high background levels from recombinant hFVIII (rhFVIII) administered before biopsy to minimize bleeding risk.

**Fig. 4:**
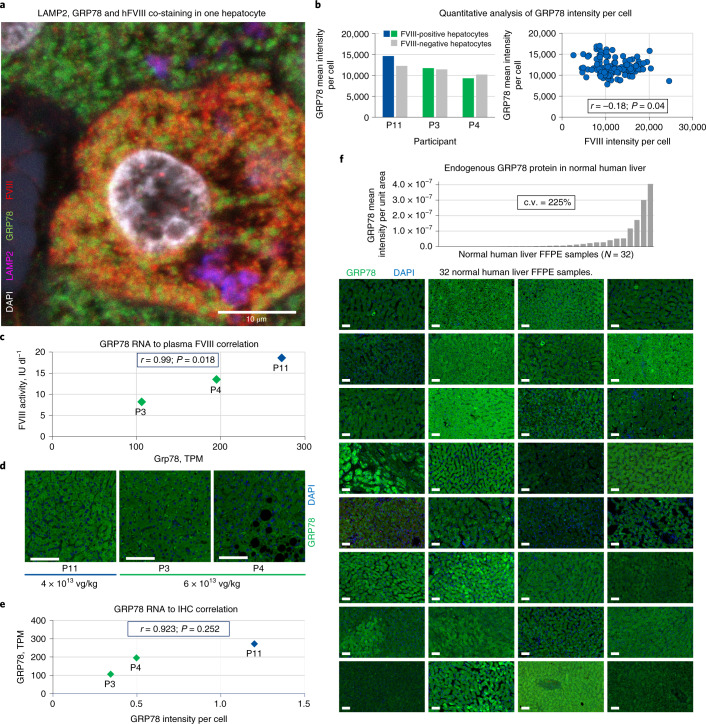
Expression of GRP78, a regulator of unfolded protein response, in human liver. **a**, Representative confocal image of LAMP2, GRP78 and hFVIII protein co-staining from one hepatocyte showing FVIII staining both in the lysosome compartment (indicating FVIII being taken up via the endocytic pathway) and in the ER compartment (FVIII protein expressed from AAV5-hFVIII-SQ). The image is from participant 4. Confocal images were acquired using a Leica TCS Sp8 white-light laser and HyD detectors at 1,024 × 1,024 pixels with an output at 300 ppi. Images were cropped and scale bars added. No additional editing was performed. Representative images of LAMP2, GRP78 and hFVIII protein co-staining from all participant biopsy samples and one normal liver are shown in Extended Data Fig. 7. **b**, Quantitative analysis of GRP78 intensity per cell (expressing hFVIII-SQ or not in the ER) and correlation between GRP78 intensity per cell and FVIII intensity per cell (Pearson correlation coefficient, *r* = −0.18; *P* = 0.04, two-tailed; total of 134 cells from participants 3 (28 cells), 4 (40 cells), 15 (16 cells) and 11 (50 cells), examined over one slide). **c**, Correlation between GRP78 RNA levels and plasma FVIII activity in the three participants with detectable plasma FVIII activity (Pearson correlation coefficient, *r* = −0.99; *P* = 0.018, two-tailed). Participants 1 and 15 had extremely low levels of FVIII RNA and thus very low/undetectable plasma FVIII activity and are therefore not included in this plot. **d**, GRP78 protein staining by IHC in participants 3, 4 and 11 (representative images from one section per participant). Images were captured at 2,048 × 2,048 pixels and output at 300 ppi. Scale bars, 100 µm. **e**, Correlation between liver GRP78 RNA and liver GRP78 IHC signals (Pearson correlation coefficient, *r* = 0.923; *P* = 0.252, two-tailed; *n* = 25,887 cells over one slide (participant 3), *n* = 37,663 cells over one slide (participant 4) and *n* = 9,091 cells over one slide (participant 11)). **f**, IHC of endogenous GRP78 protein in 32 normal human liver FFPE samples (one section per individual). Images were captured at 2,048 × 2,048 pixels and output at 300 ppi. Scale bars, 50 µm. Source data

**Extended Data Fig. 7:**
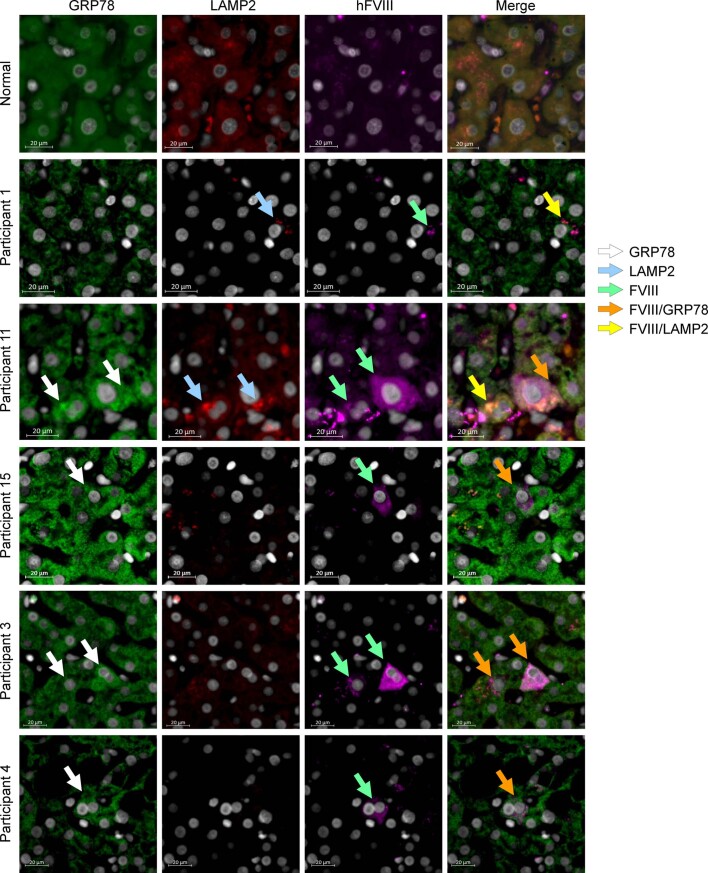
Detection of hFVIII protein in human liver and co-localization with organelle-specific markers for lysosomes (LAMP2) and endoplasmic reticulum (GRP78). LAMP2, GRP78 and hFVIII protein co-staining of hepatocytes from study participant biopsies and one normal liver. Images shown are representative of one section per study participant; the entire biopsy section was reviewed to evaluate hFVIII, LAMP2 and GRP78 co-localization. Images were captured at 2048 × 2048 pixels and output at 300 ppi. LAMP2, lysosome-associated membrane glycoprotein 2; GRP78, glucose-regulated protein 78.

Thus, a more focused analysis was required to measure valoctocogene roxaparvovec-expressed hFVIII-SQ protein. Clearance of rhFVIII occurs mainly through the liver, via two parallel linked pathways: rhFVIII complexed with von Willebrand factor is cleared mainly by Kupffer cells, while the minor unbound rhFVIII fraction is cleared by hepatocytes^27^, presumably by endocytosis and catabolism in the lysosomes.^28^ Vector-expressed hFVIII-SQ protein is expected to be detected in the endoplasmic reticulum (ER) compartment of the hepatocytes.^3,29^ Therefore, IHC analyses of hFVIII colocalizing in hepatocyte lysosomes or ER using confocal microscopy were performed, using LAMP2 (lysosome-associated membrane protein 2) and GRP78 (glucose-regulated protein 78) as organelle-specific markers for lysosomes and ER, respectively. hFVIII-SQ protein expressed from valoctocogene roxaparvovec vector genome colocalized with GRP78 in the ER, while hFVIII protein taken up by hepatocytes colocalized with LAMP2 in the lysosomes (Fig. 4a and Extended Data Fig. 7).

In addition to serving as an ER-specific marker, GRP78 directly interacts with human FVIII protein, aiding its folding and secretion,^29^ and elevated levels of GRP78 are indicative of ER stress.^30,31^ Studies in mice have shown that hepatocytes expressing B-domain-deleted FVIII (from vectors incorporating a stronger hepatic-specific promoter than is used in valoctocogene roxaparvovec) may mount an unfolded protein response, potentially inducing ER stress.^32–34^ To investigate the possibility of ER stress in our samples, fluorescence signals for GRP78 and FVIII protein colocalized in ER were quantified on a per-cell basis. Within individual samples, hepatocytes with detectable hFVIII-SQ protein staining in the ER did not have elevated expression of GRP78 compared to hepatocytes without hFVIII-SQ staining in the ER (Fig. 4b), suggesting that hFVIII-SQ protein expression derived from valoctocogene roxaparvovec did not elicit ER stress in these cells.

### Factors associated with level of plasma hFVIII-SQ protein activity

Among the three participants (3, 4 and 11) who had detectable plasma FVIII activity at biopsy, higher plasma FVIII activity was, surprisingly, associated with lower RNA expression (Extended Data Fig. 4e). Because of its role in directly aiding the folding and secretion of FVIII^29^, GRP78 was investigated as a factor that might contribute to this negative correlation between RNA and protein levels by its role in increasing the efficiency of folding and secretion of the hFVIII-SQ protein. Participants’ plasma hFVIII-SQ protein levels correlated with levels of both overall GRP78 RNA (*r* = 0.99, *P* = 0.018; Fig. 4c) and GRP78 protein (*r* = 0.923, *P* = 0.252; Fig. 4d,e) in the liver. IHC analysis of GRP78 in 32 normal human liver FFPE samples showed highly variable hepatocyte GRP78 expression (coefficient of variation (c.v.) = 225%; Fig. 4f), supporting the hypothesis that levels of endogenous GRP78 in the hepatocytes may play a role in variability of transgene expression in AAV5-hFVIII-SQ-treated individuals. A causative relationship, however, has not been demonstrated and is the subject of ongoing mechanistic studies. In exploratory analyses, we utilized the gene expression profiles to identify other pathways potentially contributing to higher plasma FVIII activity (Supplementary Fig. 6).

## Discussion

AAV-based gene transfer has made rapid advances in the clinic over the past several years. Two US Food and Drug Administration-approved products are now on the market^35,36^ and, as of August 2021, approximately 30 phase 3 trials of AAV gene therapies have been registered worldwide.^37^ However, at the molecular level, much remains unknown.

This liver biopsy investigation demonstrated that a single intravenous infusion of valoctocogene roxaparvovec (AAV5-hFVIII-SQ) administered to adults with severe hemophilia A resulted in transduction of human liver throughout tissues sampled without zonal bias within hepatic lobules. No clinically relevant inflammation or adverse histopathology was observed in any of the samples. Full-length circular vector genomes were present in both monomeric and concatemeric forms, primarily in the H–T configuration, 2.6–4.1 years after administration. In general, higher levels of hFVIII-SQ DNA and hFVIII-SQ RNA were observed at higher doses of AAV5-hFVIII-SQ. Interindividual variability points to a number of potential mechanisms that regulate RNA and protein expression.

Data on patterns of hepatic transduction of AAV vectors in humans have been lacking^38^. In animals, there is evidence that transduction patterns may be species and capsid dependent^38^ and coincide with lobular distribution of AAV uptake receptors (Supplementary Fig. 2a). We demonstrated unbiased distribution of AAV5-hFVIII-SQ vector genomes in human liver biopsy samples, similar to that observed in NHPs (Supplementary Fig. 7) and consistent with an even distribution of AAV5 uptake receptors in normal human liver (Supplementary Fig. 2b).

Importantly, circularization of episomal genomes was evident in transduced hepatocytes 2.6–4.1 years after gene transfer, with ≥3 copies of full-length circular vector genomes per diploid genome and approximately 50% of hepatocyte nuclei staining positive for transgene DNA in the 6 × 10^13^ vg per kg body weight group. Multiple preclinical studies demonstrating lung, liver and muscle transduction by AAV gene therapy in mice and NHPs have shown that circularized monomeric and concatemeric episomes are the major DNA species associated with long-term, persistent expression of the gene product in the target cell^8–16^. In nonclinical studies of AAV5-hFVIII-SQ^17^, circularized full-length episomes were evident in NHP and mouse livers up to 6 months after vector administration and correlated significantly with hFVIII-SQ RNA. In both humans and animals, there were dose-dependent trends in the numbers of hepatocytes transduced with vector genomes, in quantities of circular full-length/ITR-fused vector genomes, and in expression of FVIII-SQ RNA.

In the absence of serial human biopsy samples in our study, evidence from nonclinical studies of AAV5-hFVIII-SQ provides some insight into the possible kinetics of genome processing. In AAV5-hFVIII-SQ-treated mice, despite a decline in overall hFVIII-SQ vector genomes, full-length circular episomes formed in the liver as early as 1 week after dose and continued to increase through 6 months (the last time point sampled), consistent with the genome processing model described in Supplementary Fig. 3 (ref. ^17^). FVIII activity following valoctocogene roxaparvovec administration in the phase 1/2 study generally increased over the first 20–28 weeks leading to peak FVIII levels in the first year, and declined in subsequent years^5^ with a shallower rate of decline beyond year 2. Longer-term AAV5-hFVIII-SQ-derived FVIII protein expression is expected to be mediated through vector genomes in the hepatocytes, and it is encouraging that our results demonstrate the presence of stable circularized vector genome DNA in hepatocytes retrieved up to 4 years after vector administration, associated with sustained hFVIII expression. Mechanisms mediating the decline in FVIII expression could include episomal genome loss over time due to additional cellular metabolism or hepatocyte turnover, and/or epigenetic regulation/silencing of genomes. Even with the decline in FVIII over time and despite variability in response kinetics, long-term hemostatic efficacy has been maintained over 4 to 5 years of follow-up in all participants (*n* = 13) who received 4 × 10^13^ vg or 6 × 10^13^ vg per kg body weight^5,6^.

Collectively, these data suggest that long-term FVIII expression following AAV5-hFVIII-SQ transduction is associated with formation of circular episomes in the liver. However, it is possible that integrated full-length genomes can also mediate long-term expression. Low-frequency integration of both complete and partial recombinant AAV5 genomes into the host cell genome has been characterized in dog and NHP tissues and clinical liver biopsy samples^39,40^. In dogs with hemophilia A that demonstrated persistent liver-derived FVIII expression and correction of bleeding phenotype for >10 years following AAV-canine-FVIII-SQ gene transfer, <5% of remaining detected vector DNA was integrated in dog liver genomes^41^; the same study showed that plasma FVIII activity was associated with hepatocyte levels of full-length episomes^17,42^.

Unexpectedly, plasma FVIII activity was negatively associated with hFVIII-SQ RNA expression in the three responders. This may imply inefficient protein translation, folding, posttranslational modification and/or secretion, and the endogenous capacity of cells to fold and secrete protein may be a factor. B-domain-deleted FVIII transgenes produce FVIII-SQ protein that is inefficiently folded and secreted from the ER^43,44^; FVIII-SQ without codon optimization has also been shown to be inefficiently folded in the ER^29,43^. It is possible that human hepatocytes, which do not normally express FVIII, might be reaching capacity to fold and secrete hFVIII-SQ protein. It is reasonable to speculate that individuals who have a greater intrinsic ability to ‘process’ FVIII protein would be more successful at secreting FVIII into the circulation. Indeed, endogenous liver GRP78 levels appear to correlate with circulating hFVIII-SQ protein levels in mice^34^, as well as in the three participants with detectable plasma FVIII activity in our study. With a limited set of naïve human liver samples, a wide range of hepatic GRP78 expression was observed. Thus, intrinsic levels of hepatic GRP78 may contribute to the observed interindividual variability. Intraindividual variation of FVIII activity over time may also be driven in part by adaptive signaling processes in response to changes in unfolded protein or other cellular stresses^45–47^ and differential expression of genes involved in protein translation and unfolded protein-binding/chaperones pathways (Supplementary Fig. 6). Larger sample sets and mechanistic studies are needed before conclusions can be made.

In this study, we showed at the single-cell level that hepatocytes expressing hFVIII-SQ protein did not have elevated levels of GRP78 protein compared to neighboring hepatocytes that had no detectable hFVIII-SQ protein in the ER, suggesting no induction of ER stress. As our biopsy samples were taken at a single time point up to 4.1 years after vector administration, we cannot exclude the possibility that higher FVIII activity levels at earlier time points may have been associated with higher GRP78 intensity and possible induction of ER stress in the hepatocytes expressing hFVIII-SQ.

This study has some limitations. Human liver biopsy sample numbers were limited by the very small AAV5-hFVIII-SQ-dosed study population who volunteered to give biopsy samples, and no pre-dose biopsy samples were available. To definitively demonstrate how genome metabolism contributes to transgene expression patterns, biopsy samples taken during peak, steady-state and declining expression are needed; the possibility of obtaining serial biopsy samples in clinical protocols is being explored. The use of two different liver biopsy methods among the five study participants was not considered to have an important impact on the study findings. Based on known principles of human circulation, a relatively uniform degree of hepatic vector distribution and AAV transduction is anticipated following intravenous infusion, and consistent transduction levels shown across lobular regions in NHPs support this (Supplementary Fig. 8). However, we cannot state conclusively that the small number of human liver biopsy samples examined in our study are representative of the entire liver.

Importantly, all participants were clinically stable, with no reported long-term hepatic issues to date. Histopathological findings showed minimal changes that were considered by an independent expert liver pathologist not to be clinically meaningful. It is important to note, however, that the study population was young and otherwise healthy and did not have abnormal histology, for example, secondary to chronic hepatitis C, which might be present in some older patients with hemophilia. The occurrence of mild steatosis among most biopsy samples was representative of the expected high prevalence within normal male populations in developed countries^48–51^. The presence of minimal portal and lobular sinusoidal infiltrates is not unusual in liver biopsy samples, even with normal enzyme markers^19^.

In this study, long-term expression of hFVIII following a single valoctocogene roxaparvovec (AAV5-hFVIII-SQ) infusion was associated with the presence of circularized full-length genomes, with observable dose-dependent trends in the number of hepatocytes containing vector genomes, quantities of circular full-length/ITR-fused vector genomes and hFVIII-SQ gene expression in the liver. Interindividual variability in transgene expression following effective vector genome transduction may result from differences in expression of regulatory molecules involved in transcription and protein folding/secretion. Further investigation into these complex mechanisms is warranted.

## Methods

### Valoctocogene roxaparvovec (AAV5-hFVIII-SQ) vector structure

The structure, formulation and manufacturing of valoctocogene roxaparvovec has been described in detail^3,4^. Briefly, the recombinant, replication-incompetent AAV5 gene therapy vector contains a single-stranded, codon-optimized, B-domain-deleted human *F8* gene (hFVIII-SQ) controlled by a liver-specific promotor and with a synthetic polyadenylation sequence. The total vector genome is >4.9 kb in length and is flanked by AAV2-derived double-stranded ITRs at the 5′ (head, H) and 3′ (tail, T) ends. A full-length vector genome unit is defined as a vector genome containing the 5′ ITR-D region, HLP promoter, the hFVIII-SQ transgene, the synthetic polyadenylation signal and 3′ ITR-D region.

### Clinical study design

The protocol of the phase 1/2 valoctocogene roxaparvovec (AAV5-hFVIII-SQ) dose escalation, safety and efficacy study (NCT02576795) is available at https://www.nejm.org/doi/suppl/10.1056/NEJMoa1908490/suppl_file/nejmoa1908490_protocol.pdf and was approved by South Central – Oxford A Research Ethics Committee, Bristol Research Ethics Committee Centre; the study was carried out in accordance with relevant national regulations, the International Committee for Harmonisation Guidelines for Good Clinical Practice, and the principles of the Declaration of Helsinki. All participants provided written informed consent. Separate consent was sought for participation in the liver biopsy substudy. Participants received compensation for pre-biopsy and biopsy visits in the form of reimbursement to cover their expenses and time. The design of this study, its primary results and long-term safety and efficacy results up to 3 years have been published in detail^4,5^. Briefly, 15 adult male participants with severe hemophilia A at five sites in the United Kingdom were sequentially enrolled in four dose cohorts to receive a single infusion of valoctocogene roxaparvovec at doses of 6 × 10^12^ vg per kg body weight (one participant), 2 × 10^13^ vg per kg body weight (one participant), 6 × 10^13^ vg per kg body weight (seven participants) and 4 × 10^13^ weight (six participants). Participants were hospitalized and monitored for 24 h after receiving the infusion. Participants in the cohort who received a dose of 6 × 10^13^ vg per kg body weight and in the cohort who received a dose of 4 × 10^13^ vg per kg body weight received a tapering course of daily corticosteroids either prophylactically starting 3 weeks after vector infusions, or if ALT levels increased to 1.5 times or more above baseline according to the protocol^5^. Exclusion criteria included liver dysfunction defined as ALT, bilirubin or alkaline phosphatase ≥3 times the upper limit of normal, previous liver biopsy within 3 years with evidence of fibrosis, liver cirrhosis of any etiology assessed by ultrasound, and hepatitis B and hepatitis C infection.

### Liver biopsy substudy

All participants who were at least 1 year after infusion of valoctocogene roxaparvovec were eligible to be included in the optional liver biopsy substudy, with the following exploratory objectives as defined in amendment 8 of the clinical study protocol (31 January 2019): (1) to examine the histopathology of the liver following valoctocogene roxaparvovec therapy, including assessing for possible safety findings (for example, fibrosis, fatty liver disease and lymphocytic invasion); (2) to quantify FVIII DNA, RNA and protein expression within hepatocytes; (3) to determine which forms of rAAV vector DNA are present at the time of biopsy; (4) to determine the lobular transduction pattern of valoctocogene roxaparvovec in human liver (that is, periportal hepatocytes and central vein hepatocytes). Participation was entirely voluntary. Five participants consented to take part in the substudy and were enrolled between July 2019 and February 2020 at three hemophilia centers in the United Kingdom. An ultrasound examination of the liver was performed 3 months before biopsy to ensure there were no pathological findings that might interfere with a safe biopsy procedure, and a FibroScan of the liver was performed within 1 week of the biopsy to correlate with any potential histopathological findings. Participants were admitted to hospital on the morning of the procedure and were to be discharged on the following day. To ensure the safe performance of the biopsy, all participants were required to have a FVIII activity level of at least 50 IU dl^−1^ (or higher, at the investigator’s discretion) determined from blood samples drawn in the morning of the procedure. All participants received exogenous recombinant FVIII to meet this target, at doses that ranged from 3,000 to 5,000 IUs. In practice, where a calculated dose amounted to less than a full vial of FVIII, the whole vial was given to avoid wastage, and thus target levels were exceeded.

Participants underwent a transjugular or ultrasound-guided percutaneous liver biopsy performed according to the standard procedures at their institution (Table 1). The biopsy procedure was performed to obtain one core of at least 1 mm × >2 cm. A portion at least 1.0–1.2 cm long was fixed in 10% neutral buffered formalin for 48 h at ambient temperature and then paraffin-embedded (FFPE) per the local site procedure, and the remaining liver core was flash frozen in liquid nitrogen or an ice/ethanol bath for biochemical analyses. Frozen samples were shipped to the sponsor on dry ice and stored at −80 °C, and FFPE samples were shipped and stored at ambient temperature. All samples for histopathological and molecular analyses were derived from the same single biopsy specimen, for each participant.

Normal liver samples from healthy donors were sourced from the following commercial providers of de-identified human biospecimens. Each provider confirmed that tissues had been collected with informed consent for their use for research purposes: AMSBIO, BioIVT, Cureline, Discovery Life Sciences, Dx Biosamples, iSpecimen and US Biolab.

### Histopathology methods

Preparation and histopathological examination of FFPE samples was performed by pathologists at the local sites per standard local procedures, and final review and interpretation was performed centrally by an independent board-certified pathologist specialized in liver diseases. The review was performed without the presence of clinical information and in a blinded fashion. When available, on-slide controls were reviewed for comparison. Evaluation of liver biopsy samples was made with reference to published scoring/staging systems for dysplasia, nonalcoholic fatty liver disease^52^, and inflammation and fibrosis^53^. Slides were reviewed by physical glass microscopy and secondarily by digitally scanned versions of the biopsy samples. H&E was used to stain for nuclei and cytoplasm. Periodic acid-Schiff stain was used to detect glycogen storage or α1-anti-trypsin (A1AT) globules, and diastase-Periodic acid-Schiff was used to remove glycogen and enhance detection of A1AT globules. Masson’s trichrome was used to stain for type 1 collagen to detect the presence and distribution of reactive fibrosis resulting from liver injury, orcein was used to stain for copper-binding protein and hepatitis B antigen, and Perl’s stain was used to detect hemosiderin, an insoluble form of iron. Slides were imaged on a Leica Aperio AT2 DX whole-slide scanning system at ×20 and were analyzed on QuPath v0.2.3.

### In situ hybridization methods to detect AAV5-hFVIII-SQ vector DNA and RNA

FFPE liver sections of 5 µm were prepared for ISH of hFVIII-SQ DNA and RNA using a Ventana Discovery Ultra Autostainer, an RNAscope Universal 2.5 Reagent kit Brown (DNA) or RNAscope Universal 2.5 Reagent kit Red (RNA) (Advanced Cell Diagnostics), and custom-generated FVIII DNA and RNA probes. Three naïve human liver samples were also prepared as negative controls. Preservation of nucleic acids following tissue processing was confirmed using probes for expression of the housekeeping gene ubiquitin C (*UBC*); negative samples were considered to have poor nucleic acid quality and were discarded. Samples were screened for contamination or poor fixation using probes for the bacterial gene *dapB*; positive samples were excluded from further analysis. Sections were dehydrated, mounted on slides using Permount mounting medium (VWR). For DNA ISH analysis, slides were imaged on a Leica DM5000 microscope using transmitted light imaging settings, a ×20 0.75 HCX Plan Fluor objective, and a DFC550 camera. For each liver section, >10 images were captured that spanned ≥50% of the biopsy: 11 images per biopsy for participants 1 and 11, and 27–28 images per biopsy for participants 3, 4 and 15, from whom the biopsy tissue area was larger. Total hepatocyte nuclei per image were counted and scored positive for hFVIII-SQ DNA if any brown foci were observed within the nucleus. For RNA analysis, slides were imaged on a Zeiss Axio Scan.Z1 using a Plan-Apochromat ×40/0.8 objective equipped with a Hamamatsu Orca Flash camera. Image analysis was performed using Visiopharm v2020.09 to quantify hepatocytes stained positive for cytoplasmic FVIII RNA signal and the number of RISH foci per cell.

### Immunohistochemistry methods to detect hFVIII-SQ protein

Hepatic expression and distribution of hFVIII-SQ protein, along with the ER stress marker GRP78 and lysosomal marker LAMP2, were measured by IHC. FFPE livers were sectioned at 5 µm in thickness on Superfrost plus slides. Slides were deparaffinized and rehydrated in a series of decreasing graded ethanols. Antigen retrieval solution CC1 (Ventana Discovery) was used to retrieve antigen at 95 °C for 32 min. Sections were blocked in 2% normal donkey serum, 0.1% BSA and 0.3% Triton in 1× tris-buffered saline (TBS) for 45 min at room temperature. Sections were immunostained with anti-FVIII antibody (1:500 dilution; Abcam, ab139391), anti-GRP78 antibody (1:1,000 dilution; Cell Signaling Technology, C50B12) and anti-LAMP2 (1:100 dilution, Abcam, ab25631) diluted in Ventana Reaction Buffer (Ventana Medical Systems, 950-300). Slides were incubated overnight at 4 °C. Slides were washed in 3× TBS. Anti-hFVIII antibody was detected using donkey anti-sheep IgG (H + L) cross-adsorbed secondary antibody conjugated to Alexa Fluor 647 (1:1,000 dilution; A-21448, Thermo Fisher Scientific). Anti-GRP78 antibody was detected using donkey anti-rabbit IgG (H + L) highly cross-adsorbed secondary antibody conjugated with Alexa Fluor 555 (1:1,000 dilution; A-21206; Thermo Fisher Scientific). Anti-LAMP2 antibody was detected using donkey anti-mouse IgG (H + L) cross-adsorbed secondary antibody conjugated to Alexa Fluor 488 (1:500 dilution; A-31570, Thermo Fisher Scientific). All secondary antibodies were diluted in Ventana Reaction Buffer and sections were incubated for 1 h at room temperature. Slides were washed in 1× TBS, counterstained with DAPI and mounted with Fluoromount G. Slides were imaged on a Zeiss Axio Scan.Z1 using a Plan-Apochromat ×20/0.8 objective equipped with a Hamamatsu Orca Flash camera. Image analysis was performed using Visiopharm v2020.09. Hepatocytes were scored as either positive or negative for hFVIII protein. Mean signal intensity of hFVIII and GRP78 was measured in every hepatocyte across the region analyzed.

### Molecular methods for quantifying hFVIII-SQ genome forms

Molecular methods are explained in detail below. In summary, quantitative measurement of hFVIII-SQ vector genome forms was performed by treating isolated liver DNA with various DNA digestion enzymes followed by ddPCR assays using different custom-generated primers/probe sets: the ITR fusion assay measures 5′ to 3′ ITR recombination (that is, H–T); the R1–R11-linked assay measures full-length hFVIII-SQ genomes capable of giving rise to stable hFVIII-SQ transcript (although there is a possibility that some genomes might contain the R1 and R11 amplicon target regions via H–T ITR fusion, but may be missing the central portion of the transgene); the R2–R10 assay measures linked genomes slightly more distal to ITRs; and the SQ assay measures overall vector genomes (full length and fragments; Supplementary Fig. 4). PS-DNase digests linear DNA, including genomic DNA, while leaving circular genomes intact. KpnI, a DNA restriction enzyme that cuts within hFVIII-SQ vector genome at nucleotide positions 2,207 and 3,596, separates out vector genome units within the concatemeric forms. The separated vector genome units can be captured within individual droplets, enabling quantification of genome units within concatemeric vector genomes.

### DNA enzyme treatment procedures

DNA was extracted using AllPrep DNA/RNA micro kits (Qiagen) following the manufacturer’s instructions from participant liver biopsy samples and naïve, control human liver samples (iSpecimen). Samples were digested with the restriction enzyme KpnI, which cuts the vector genome at nucleotide positions 2,207 and 3,596, to separate out vector genome units within concatemeric forms (Supplementary Fig. 4c). Before the droplet generation step of ddPCR, 5 µl of DNA at a concentration of 2 ng µl^−1^ was incubated with 4 units of high-fidelity KpnI (KpnI-HF) enzyme (New England Biolabs) in the ddPCR reaction mixture at 37 °C for 30 min.

An additional set of samples was digested with PS-DNase (Lucigen) to hydrolyze all linear forms of DNA and isolate circular DNA. It is important to note that the use of PS-DNase to eliminate linear genomes results in an underestimation of the amount of circular genomes because: (1) the DNA extraction process is likely to shear/linearize large concatemeric circular episomes, rendering them sensitive to PS-DNase digestion (the extent to which this occurs is unclear); and (2) PS-DNase has been shown to degrade 25–35% of circular plasmid DNA^20^. Thus, the DNA measurements after PS-DNase treatment should be regarded as minimum numbers. The PS-DNase treatment nevertheless results in a very strong enrichment of circular species, with >99% degradation of genomic DNA targets (Supplementary Table 2), while most circular vector genomes survive. Before droplet generation, 200 ng of total DNA was incubated for 16 h at 37 °C with 50 units per µg of PS-DNase in 33 mM Tris-acetate (pH 7.5), 66 mM potassium acetate, 10 mM magnesium acetate, 0.5 mM dithiothreitol and 1 mM adenosine triphosphate. PS-DNase was then inactivated with a 20-min incubation at 80 °C, and samples were diluted to 2 ng µl^−1^ in 10 mM Tris-Cl (pH 8.5) and 0.05% Pluronic F-68. For the ddPCR reactions, 5 µl of dilute sample was used.

ddPCR was also performed with samples treated with both PS-DNase and KpnI restriction enzymes to quantify individual vector genome units within circular concatemers (Supplementary Fig. 4c). DNA samples were treated with PS-DNase, heat inactivated and diluted as described above. Before the droplet generation step, 4 units of KpnI-HF enzyme were added to the ddPCR reaction mix and incubated at 37 °C for 30 min.

### ddPCR procedures to detect vector genomes and inverted terminal repeat fusions

Quantities of vector genome forms in samples were measured with ddPCR, which captures individual DNA molecules in thousands of water–oil emulsion droplets before PCR-mediated amplification within each droplet and discrete measurement of endpoint fluorescence of individual droplets. Each droplet is counted as negative or positive by fluorescence, and Poisson statistics are applied to the fraction of positive droplets to estimate the copy number of target DNA molecules per sample. In this analysis, a variant of ddPCR called drop-phase ddPCR was performed to detect and quantify the levels of paired target sequences together on a single DNA molecule to measure the contiguity of the DNA molecule, using two different fluorescent tags (FAM and HEX; Supplementary Fig. 4a). The number of double-positive droplets was then calculated and the total copy number of molecules with both target sequences was estimated using the software QuantaSoft v1.7.4.0917 (Bio-Rad), which includes an algorithm that accounts for the probability that some double-positive droplets may occur due to chance. ddPCR of the endogenous gene adaptor-related protein complex 3, beta 1 subunit (*AP3B1*) was also performed to provide a normalization reference for calculating vector copy numbers for each diploid genome from the concentration of droplets containing Hex fluorophores. For all molecular analyses involving ddPCR, at least three technical replicates were used per sample, per condition.

Vector genome ITR fusions were measured using ddPCR technology, as described above. The primer and probe sets used for ddPCR and drop-phase ddPCR are presented in Supplementary Fig. 4b and Supplementary Table 1. To specifically detect ITR fusions, two directional PCR primers were designed: fusions of the 5′ and 3′ (H–T) ITRs were detected using forward and reverse primers located on the 5′ and 3′ ends of the linear genome that extend toward the ITR and only produce an amplicon when 5′ and 3′ ITRs are fused.

Full-length vector genomes capable of giving rise to stable hFVIII-SQ transcription were detected with the R1–R11 linkage assay. Drop-phase ddPCR reactions were used to identify the co-occurrence of amplicons of R1 and R11, which overlap with the D segments of the ITR on the 5′ and 3′ ends of the genome, respectively (Supplementary Fig. 4b). To capture circularized genomes with rearrangements or deletions in the ITRs, drop-phase ddPCR using R2 and R10 amplicons that are slightly more distal to the ITR was also performed (Supplementary Fig. 4b).

Following treatment with KpnI (to separate individual vector genome units within circular concatemers) and/or PS-DNase (to eliminate linear forms), 10 ng of DNA was used in each ddPCR reaction. The reaction mixture contained 1′ ddPCR Supermix for Probes without deoxyuridine triphosphate (Bio-Rad), 250 nM each of forward and reverse primers, 900 nM of probes and 5 µl of sample for a final reaction volume of 25 µl. A Bio-Rad Auto Droplet Generator was used to generate droplets from the reaction mix and QX200 Droplet Generation Oil for Probes (Bio-Rad), which were then transferred into a 96-well plate. PCR was performed in a C1000 Touch Thermal Cycler (Bio-Rad) as follows, for all primer sets except for the ITR fusion assay: 10 min at 95 °C, 40 cycles of 30 s at 95 °C and 1 min at 58 °C, 10 min at 98 °C, and hold at 4 °C. H–T ITR amplification cycle conditions were 40 cycles of 30 s of denaturation at 94 °C, 35 s annealing at 59 °C and 65 s extension at 72 °C. Samples were read using a QX200 droplet reader (Bio-Rad) and the total concentration of target sequences and linked copies of target sequences were processed with QuantaSoft software v1.7.4.0917 (Bio-Rad).

### Southern blot procedures to characterize circular episomes

Southern blotting was used to identify the configuration of circular episomes. For each sample, 5 µg of DNA was digested for 2 h with 50 units each of EcoRI and HindIII restriction enzymes (New England Biolabs), which do not cut within vector genomes, followed by PS-DNase treatment at 37 °C for 16 h. The reaction was halted by heat inactivation at 80 °C for 20 min; an additional set of samples was also incubated at 37 °C for 2 h with 10 units of KpnI. For all samples, DNA was isolated with phenol-chloroform extraction, precipitated in ethanol and resuspended in 30 µl of nuclease-free water. Purified DNA samples were mixed with 6× gel loading dye (New England Biolabs), loaded into a 0.7% agarose gel containing 0.5× SYBR Safe DNA Gel Stain dye (Thermo Fisher Scientific), and electrophoresis was performed at 30 to 35 V for 16 to 18 h at room temperature. DNA was depurinated in 0.5 M sodium hydroxide and 1.5 M sodium chloride denaturing buffer for 30 min at room temperature with gentle agitation, neutralized in 1.5 M NaCl and 0.5 M Tris-HCl (pH 7.0) neutralizing buffer for 30 min, and then soaked in 20× SSC transfer buffer (3 M sodium chloride and sodium citrate (pH 7.0)) for 30 min.

DNA was then transferred to a positively charged nylon membrane using a Whatman Nytran SuPerCharge TurboBlotter system (Sigma-Aldrich) and immobilized with a UV Crosslinker (VWR). Biotin-labeled probes binding to both ends of the vector genome were generated using the Biotin PCR labeling kit (PromoCell); the primer sequences used for PCR reactions are presented in Supplementary Table 3, and their location on the vector genome is shown in Supplementary Fig. 5. Southern blot hybridization was performed overnight at 65 °C using 30 ng of each biotinylated probe and 100 µg of sheared salmon sperm DNA (Thermo Fisher Scientific). A Thermo Fisher Chemiluminescent Nucleic Acid Detection Module kit was used to detect hybridization and the membrane was imaged with a ChemiDoc Imager (Bio-Rad).

### RNA quantification analysis

Total RNA and DNA was extracted from liver samples using an AllPrep DNA/RNA micro kit (Qiagen). Concentration of extracted RNA was measured using a Nanodrop 8000 spectrophotometer (Thermo Fisher Scientific) and then diluted to 15 ng µl^−1^. For each sample, 150 ng RNA was reverse transcribed to generate first-strand cDNA using SuperScript VILO Master Mix (Thermo Fisher Scientific). Control samples without cDNA were generated by omitting the reverse transcriptase from the mix. cDNA positive and negative samples were diluted to 1:5 and 1:50, respectively, with elution buffer (10 mM Tris-Cl, pH 8.5; Qiagen) containing 0.05% Pluronic PF-68 (Thermo Fisher Scientific).

ddPCR was then performed as described above using 5 µl of diluted sample and primers and probes designed to target the SQ region of the vector genome (Supplementary Fig. 4b and Supplementary Table 1). The amount of FVIII-SQ RNA detected in human liver biopsy samples was normalized to three endogenous housekeeping RNAs: YWHAZ, ACTB (β-actin) and RPLP0 (ribosomal protein lateral stalk subunit P0).

### RNA sequencing to explore mechanisms of interindividual variability

RNA-seq analyses were performed to characterize the gene expression profiles of participants’ hepatocyte RNAs via the TruSeq RNA Exome protocol (Illumina NextSeq platform). For each participant included in the analysis, a sequencing library was generated from approximately 100 ng human liver total RNA. Briefly, total RNA was fragmented followed by cDNA generation. The indexed sequencing library was constructed from the cDNA. The five indexed sequencing libraries were pooled, and the coding regions of the human transcriptome were captured with sequence-specific probes in a single hybridization reaction. The post-hybridization library was amplified with ten PCR cycles before the sequencing run. Data were analyzed by ROSALIND v3.19.0.5 (https://rosalind.onramp.bio/), with a HyperScale architecture developed by ROSALIND. Several database sources were referenced for enrichment analysis, including Interpro (PANTHER v15.0; http://www.pantherdb.org/), NCBI (https://www.ncbi.nlm.nih.gov/nuccore/), MSigDB (Molecular Signature Database v7.2; http://www.gsea-msigdb.org/gsea/index.jsp), REACTOME (Reactome database release 73; https://reactome.org/), WikiPathways (https://www.wikipathways.org/) and DAVID (The Database for Annotation, Visualization and Integrated Discovery) v6.8 (https://david.ncifcrf.gov/).^54,55^ Enrichment was calculated relative to a set of background genes relevant for the experiment.

### Statistics and reproducibility

Statistical analysis was conducted using either a two-tailed unpaired student *t*-test or calculation of the Pearson correlation coefficient using Prism (v7.01, GraphPad). The Benjamini–Hochberg method for multiple testing was used in the analysis exploring correlation of gene expression profiles and plasma FVIII activity^56^.

Due to limited amounts of available tissue samples from each participant, the opportunity to replicate experiments was restricted. For histopathology evaluation, one to four sequentially deeper histologic sections (levels) were prepared from each biopsy and were reviewed both by the local pathologist and by the central pathologist.

ISH staining to show hFVIII-SQ DNA was performed on at least ten images per biopsy, spanning ≥50% of the biopsy tissue area; quantification of cells that stained positive for hFVIII-SQ DNA was calculated as the mean across all images spanning the biopsy section. To explore differences in transgene expression between two participants who received the same dose of valoctocogene roxaparvovec, one section each from participants 11 and 15 was used to evaluate percentage of hepatocytes that stained positive for hFVIII-SQ RNA by RISH; FVIII RNA foci were quantified in 1,300–2,200 individual hepatocytes from participants 11 and 15 and presented as mean foci per cell. No technical replicates were used for statistical analysis of DNA and RISH quantification.

For histology (IHC and ISH), RNA-seq and associated statistical analyses, each experiment was performed once only due to limited quantities of tissues and RNA.

Southern blot analyses were performed to derive qualitative information about the forms and configurations of hFVIII-SQ DNA episomes; the limited available quantities of extracted DNA were sufficient to run one blot after DNA treatment with PS-DNase and one blot after PS-DNase + KpnI for each sample, but did not permit any replication of these experiments.

### Mouse study and nonhuman primate study designs

Animal study methods relating to data for cross-species comparison presented in Supplementary Figs. 2, 7 and 8 are described in full elsewhere^3,17^. Briefly, in separate experiments, *Rag2*^−/−^ *FVIII*^−/−^ double knockout mice received a single intravenous bolus tail injection of AAV5-hFVIII-SQ, and male cynomolgus monkeys that screened negative for total antibodies and neutralizing factors against AAV5 were dosed into the saphenous vein. Liver samples were taken and snap frozen at necropsy at post-dose times as reported in the results. For histopathology and ISH analysis, FFPE liver sections were collected and prepared by standard methods. ISH analysis to detect hepatocyte nuclei positive for hFVIII-SQ DNA was performed as described above.

For ISH immunostaining to evaluate the distribution of hFVIII-SQ vector genome and AAV receptors, sections were immunostained with anti-FVIII antibody (1:500 dilution; Abcam, ab139391), anti-VP3 antibody (1:1,000 dilution; NB100-93577, Novus), anti-PDGFRA antibody (1:1,000 dilution; Millipore, C50B12) and anti-AAVR (1:50 dilution; Abcam, ab105385). Anti-hFVIII antibody was detected using donkey anti-sheep IgG (H + L) cross-adsorbed secondary antibody conjugated to Alexa Fluor 647 (1:1,000 dilution; Thermo Fisher Scientific, A-21448). Anti-VP3 and anti-PDGFRA antibodies were detected using donkey anti-rabbit IgG (H + L) highly cross-adsorbed secondary antibody conjugated with Alexa Fluor 555 (1:1,000 dilution; Thermo Fisher Scientific, A-21206). Anti-AAVR antibody was detected using donkey anti-mouse IgG (H + L) cross-adsorbed secondary antibody conjugated to Alexa Fluor 488 (1:500 dilution; Thermo Fisher Scientific, A-31570). Slides were imaged on a Zeiss Axio Scan.Z1 using a Plan-Apochromat ×20/0.8 objective equipped with a Hamamatsu Orca Flash camera.

Molecular analysis was performed on separate samples; procedures for drop-phase ddPCR quantification of vector DNA copy numbers and hFVIII-SQ RNA transcripts are described above.

All in vivo animal procedures were performed in accordance with institutional guidelines under protocols approved by the Institutional Animal Care and Use Committees of the Buck Institute (mice) and the Charles River Laboratories facility (monkeys).

### Reporting Summary

Further information on research design is available in the Nature Research Reporting Summary linked to this article.

## Online content

Any methods, additional references, Nature Research reporting summaries, source data, extended data, supplementary information, acknowledgements, peer review information; details of author contributions and competing interests; and statements of data and code availability are available at 10.1038/s41591-022-01751-0.

### Supplementary information

Supplementary Information Supplementary Tables 1–3, Supplementary Figs. 1–8 and Supplementary Source Data for Supplementary Figs. 6b,c and 8.

Reporting Summary

### Source data

Source Data Fig. 1 Statistical source data (Figs. 1c,d). Unprocessed Southern blots (Fig. 1e).

Source Data Fig. 2 Statistical source data (Fig. 2a–d).

Source Data Fig. 3 Statistical source data (Figs. 3a,b).

Source Data Fig. 4 Component images for confocal image of LAMP2, GRP78 and hFVIII protein co-staining (Fig. 4a). Statistical source data (Figs. 4b,c,e,f).

Source Data Extended Data Fig. 3 Statistical source data (Extended Data Figs 3a–e). Unprocessed Southern blots (Extended Data Fig. 3f).

Source Data Extended Data Fig. 4 Statistical source data (Extended Data Figs. 4a–e).

Source Data Extended Data Fig. 5 Statistical source data (Extended Data Figs. 5a–c).

Source Data Extended Data Fig. 6 Statistical source data.

## Figures and Tables

**Extended Data Table 1:**
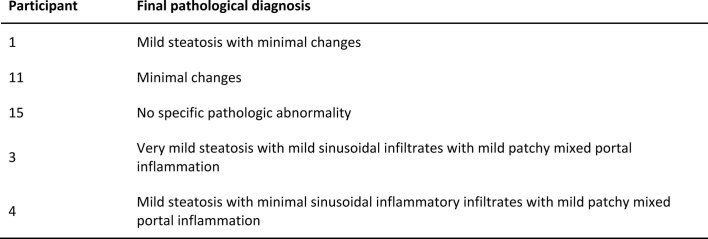
Histopathological diagnosis by a central independent liver pathology expert based on assessment of liver biopsy samples from five participants 2.6–4.1 years after gene transfer with valoctocogene roxaparvovec

## Data Availability

Due to the very small number of participants in this study, drawn from the limited number of individuals in this rare disease population, the gene expression profiles/sequencing libraries generated for each participant have not been shared via a public repository to avoid potentially compromising patients’ identities. However, the de-identified individual participant data that underlie the results reported in this article (including text, tables, figures and appendices) will be made available together with the research protocol and data dictionaries, for noncommercial, academic purposes. Additional supporting documents may be available upon request. Investigators will be able to request access to these data and supporting documents via a website (https://www.biomarin.com/) beginning 6 months and ending 2 years after publication. Data associated with any ongoing development program will be made available within 6 months after approval of the relevant product. Requests must include a research proposal clarifying how the data will be used, including proposed analysis methodology. Research proposals will be evaluated relative to publicly available criteria available at https://www.biomarin.com/ to determine if access will be given, contingent upon execution of a data access agreement with BioMarin Pharmaceutical. Source data are provided with this paper.
