# Supplementary material for: Interindividual variability in transgene mRNA and protein production following adeno-associated virus gene therapy for hemophilia A
Source: Nat Med. 2022 Apr 11;28(4):789–97. doi: 10.1038/s41591-022-01751-0 (PMC9018415; doi:10.1038/s41591-022-01751-0)
Supplement: Supplementary file 1 — Supplementary Tables 1–3, Supplementary Figs. 1–8 and Supplementary Source Data for Supplementary Figs. 6b,c and 8. [file 41591_2022_1751_MOESM1_ESM.pdf]

nature portfolio

https://doi.org/10.1038/s41591-022-01751-0

Supplementary information

Interindividual variability in transgene mRNA and protein production following adeno-associated virus gene therapy for hemophilia A

In the format provided by the authors and unedited

Contents

Supplementary Tables

Supplementary Table 1 | ddPCR primers and probes 2

Supplementary Table 2 | Host genome measurement before and after treatment with PS-DNase 3

Supplementary Table 3 | Primer sequences to generate biotinylated Southern blot probes 3

Supplementary Figures

Supplementary Fig. 1 | Schematic illustrating the process of liver-directed AAV vector administration, transduction of hepatocytes, and evolution of vector genome forms over time. 4

Supplementary Fig. 2 | Distribution of hFVIII-SQ vector genome and AAV receptors in animal livers after AAV5-FVIII-SQ gene transfer in preclinical studies, and distribution of AAV receptors in normal human liver. 5

Supplementary Fig. 3 | Model of AAV genome processing and forms associated with long-term expression. 6

Supplementary Fig. 4 | Molecular analysis of hFVIII-SQ vector genomes for quantification of circular episomal forms of hFVIII-SQ in liver biopsy samples. 7

Supplementary Fig. 5 | Locations of the Southern Blot probes on various vector genome configurations and expected restriction-digested fragment sizes during Southern blotting. 9

Supplementary Fig. 6 | Expression of molecules in the translation initiation and unfolded protein binding pathway positively correlated with FVIII activity 10

Supplementary Fig. 7 | Cross-species comparison of valoctocogene roxaparvovec transduction in hepatocytes. 11

Supplementary Fig. 8 | Distribution of hFVIII-SQ vector genome in NHP livers after AAV5-FVIII-SQ gene transfer. 12

References 13

Source data for Supplementary Figures 14

Supplementary Table 1 | ddPCR primers and probes

Drop-phase ddPCR	Amplicon	Amplicon size (bp)	Primers/ probe	Sequence (5'–3')
	ITR fusion	836[a]	Forward	CACCAGTGGACCCTGTTCTTC
			Reverse	CTGGTGGCAGAGAAGCAGAAC
			Probe (FAM)	AGCTTCACCCCTGTGGTGAACAGC
R1-R11	R1	149	Forward	CACTAGGGGTTCCTGTTTGCTG
			Reverse	AACCAAGGTCACCCCAGTTA
			Probe (HEX)	ACAGGACGCTGTGGTTTCTGAGCCAGGGGG
	R11	159	Forward	ACCAGATACCTGAGGATTCACC
			Reverse	CATCACTAGGGGTTCCTCACAC
			Probe (FAM)	CCCTGAGGATGGAGGTGCTGGGCTG
R2-R10	R2	132	Forward	TAATATTCACCAGCAGCCTCCC
			Reverse	AATCTGCATGGTGGCGATTC
			Probe (HEX)	AGGGCCCTGTCTCCTCAGCTTCAGGCACCA
	R10	109	Forward	TGGACCCTGTTCTTCCAGAATG
			Reverse	TCAGGTATCTGGTCAGCAGG
			Probe (FAM)	GGCAACCAGGACAGCTTCACCCCTGTGG
	SQ	153	Forward	ACATCATGCACAGCATCAATGGCTA
			Reverse	CCATCTTGTGCTTGAAGGTG
			Probe (FAM)	CCTGAGCATTGGGGCCCAGA

[a]If both ITRs are intact.

AP3B1, ACTB and YWHAZ primers and probes were purchased commercially from Bio-Rad (Hercules, CA) and the sequences were undisclosed.

AP3B1, adaptor-related protein complex 3 beta 1 subunit; YWHAZ, tyrosine 3-monooxygenase/ tryptophan 5-monooxygenase activation protein zeta; ACTB, beta-actin; Rplp0, ribosomal protein lateral stalk subunit P0; bp, base pairs; ddPCR, droplet digital PCR; ITR, inverted terminal repeat; PCR, polymerase chain reaction.

Supplementary Table 2 | Host genome measurement before and after treatment with PS-DNase

PS-DNase treatment resulted in 99% degradation of genomic DNA targets.

	**Host genome copies of *AP3B1* per µL of reaction**		**% Remaining of host genome**
**Samples**	**Before PS+**	**After PS+**	
Naïve	79.95	0.59	0.7%
Participant 15	69.32	0.32	0.5%
Participant 3	47.78	0.32	0.7%
Participant 4	55.80	0.31	0.6%
Participant 1	80.90	0.38	0.5%
Participant 11	86.70	0.53	0.6%

ddPCR of the endogenous gene *adaptor-related protein complex 3, beta 1 subunit (AP3B1)* was performed to provide a normalization reference for calculating vector copy numbers per diploid genome.

Supplementary Table 3 | Primer sequences to generate biotinylated Southern blot probes

**Probe**	**Length (bp)**	**Primer direction**	**Primer sequence (5'–3')**
Probe 1	620	Forward	TAATATTCACCAGCAGCCTCCC
		Reverse	ATGGGGCCATTCTCCTTCAG
Probe 2	645	Forward	AGTTCAGCAGCCTGTACATCAG
		Reverse	TCAGGTATCTGGTCAGCAGG

bp, base pairs.

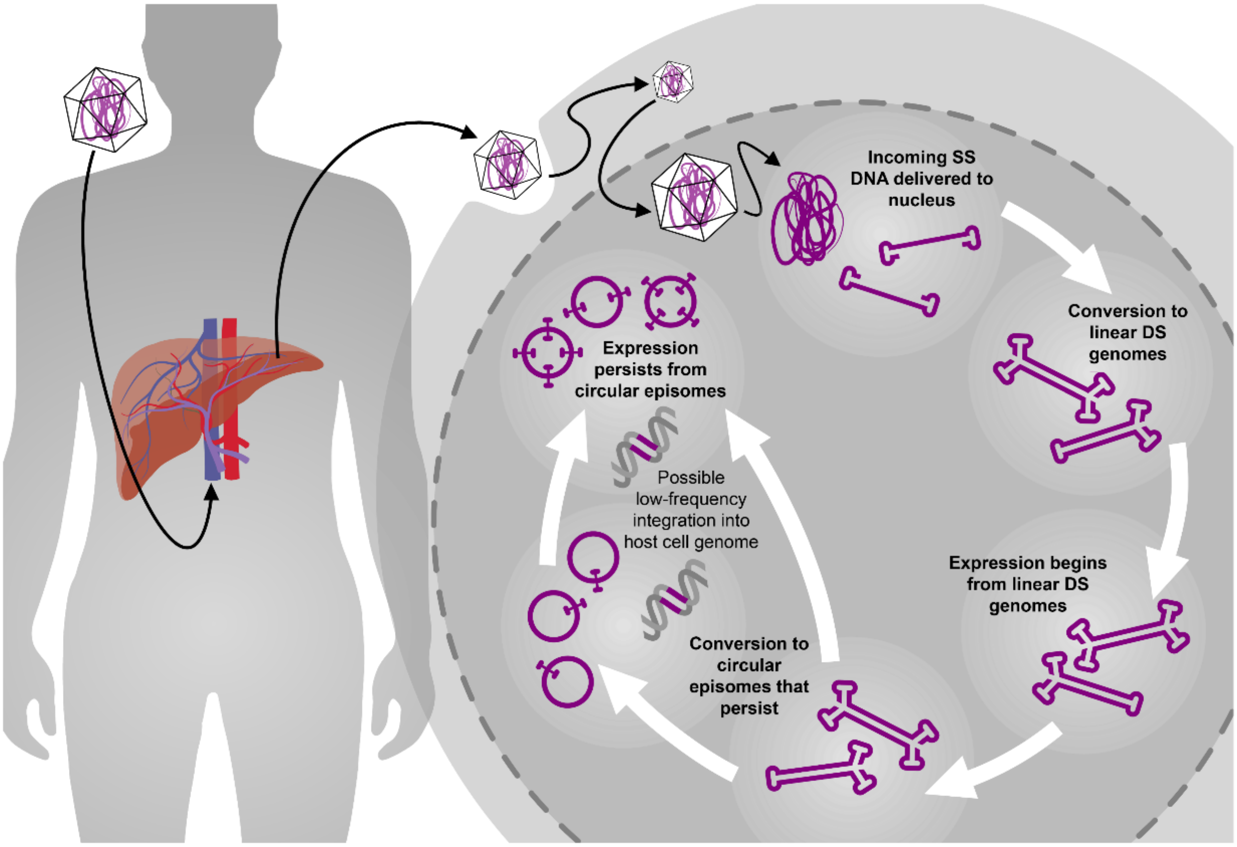

Supplementary Fig. 1 | Schematic illustrating the process of liver-directed AAV vector administration, transduction of hepatocytes, and evolution of vector genome forms over time.
Adapted from Wang et al.[1] and Sihn et al.[2]

DS, double-stranded; SS, single-stranded

a Mouse liver

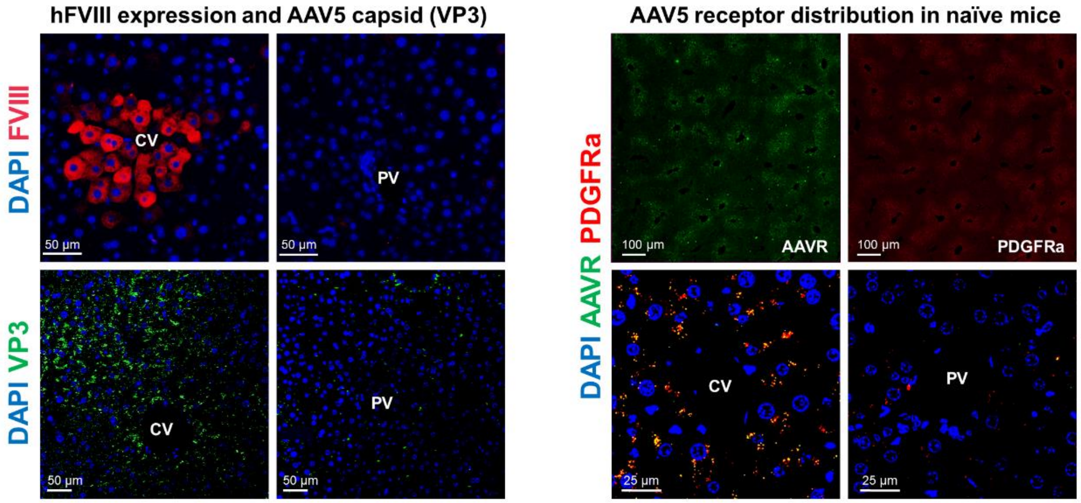

b Human liver

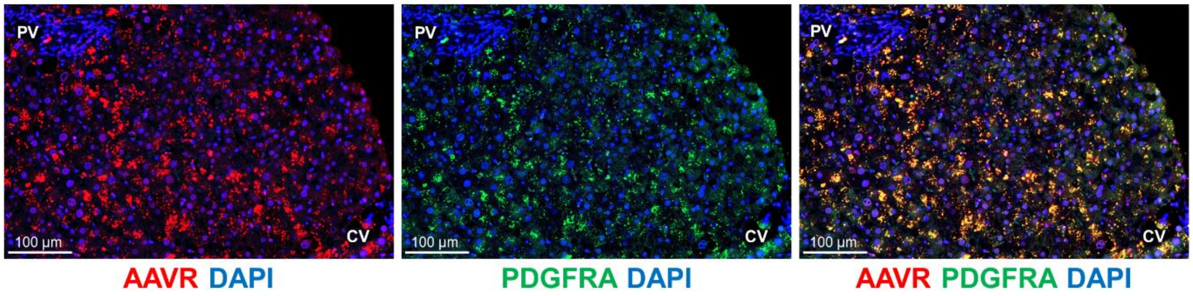

Supplementary Fig. 2 | Distribution of hFVIII-SQ vector genome and AAV receptors in animal livers after AAV5-FVIII-SQ gene transfer in preclinical studies[3], and distribution of AAV receptors in normal human liver.
a) IHC immunostaining images showing AAV5 capsids (detected using antibody against VP3 capsid protein), hFVIII-SQ protein, and AAV5 pan-receptor (AAVR) and co-receptors (PDGFRa) distributed preferentially in a peri-central pattern in liver samples from $Rag2^{-/-}$ $FVIII^{-/-}$ double knockout mice dosed with AAV5-hFVIII-SQ, by methods described by Sihn et al.[2] For FVIII and AAV5-VP3 immunostaining, images are representative of 10 liver tissues from individual animals stained (one section per animal). For AAVR and PDGRFa, images are representative of 16 liver tissues from individual animals stained (one section per animal). Images captured at 2048 × 2048 pixels and output at 300 ppi. **b)** Unbiased distribution of AAVR pan-receptor and AAV5 co-receptor (PDGFRa) in normal human liver. Images are representative of three normal human liver tissues stained (one section analyzed per individual). Images captured at 2048 × 2048 pixels and output at 300 ppi.

AAV, adeno-associated virus; AAVR, AAV receptor; CV, central vein; DAPI, 4’,6-diamidino-2-phenylindole; ddPCR, droplet digital polymerase chain reaction; PDGFRa, platelet-derived growth factor a; IHC, immunohistochemistry; PV, portal vein; AAV5-VP3, an AAV5 capsid protein.

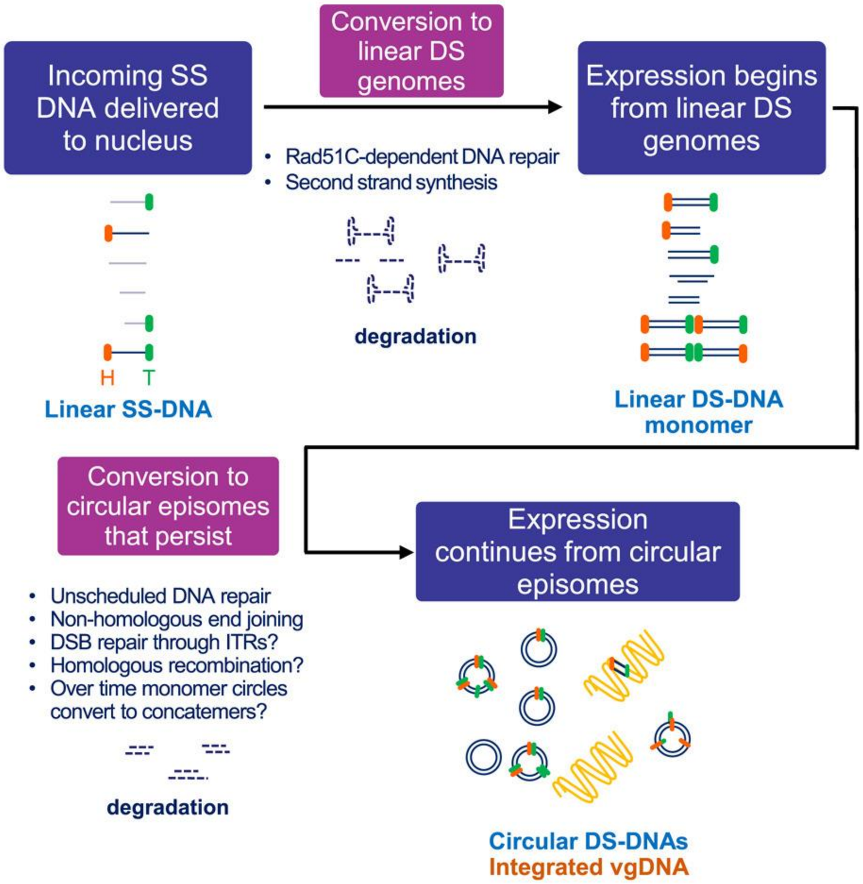

Supplementary Fig. 3 | Model of AAV genome processing and forms associated with long-term expression.
Reproduced from Sihn et al.[2] under Creative Commons license CC BY-NC-ND 4.0, ©2022 BioMarin Pharmaceutical Inc. Available at: https://www.cell.com/molecular-therapy-family/methods/fulltext/S2329-0501(21)00192-3

Multiple preclinical studies in lung, liver, and muscle transduced by AAV gene therapy in mice and NHP have shown that circularized monomeric and concatemeric episomes are the major DNA species associated with long term, persistent expression of the gene product in the target cell. The circularized episomes are formed through a complex DNA processing mechanism. Single-stranded vector DNA encapsulated in the capsids is transported into the nucleus, where much is degraded.[4,5] AAV Vector genome processing, particularly for oversized genomes, can be mediated by Rad51C, a single-stranded DNA-binding protein that promotes strand annealing in response to double strand breaks, allowing for the single-stranded vector DNA to be converted to double-stranded linear genomes via DNA repair proteins.[4–7] The resultant double-stranded linear full-length genomes can support transgene expression, but can also be degraded over time and some are converted via non-homologous end-joining into stable monomeric and concatemeric circular episomes.[7–12] Circular episomal conversion via homologous recombination is also possible through interactions with inverted terminal repeats (ITRs).[13] Although full-length AAV vector genomes (including promoter, transgene and poly A signal) might be integrated into host genomes which could in part mediate the expression of the transgene, the frequency of integration for AAV vectors in mice, rabbits, nonhuman primates, dogs, and humans have been shown to be very low (at between 1 in 1,000 to 1 in 10,000 cells), at orders of magnitude lower than seen for lentiviral vectors and the spontaneous natural human mutation rate.[10,12,14–17]

DSB, DNA double strand break; DNA-PKcs, DNA-dependent protein kinase, catalytic subunit; DS, double-stranded; ITR, inverted terminal repeat; SS, single-stranded; vg, vector genome.

a

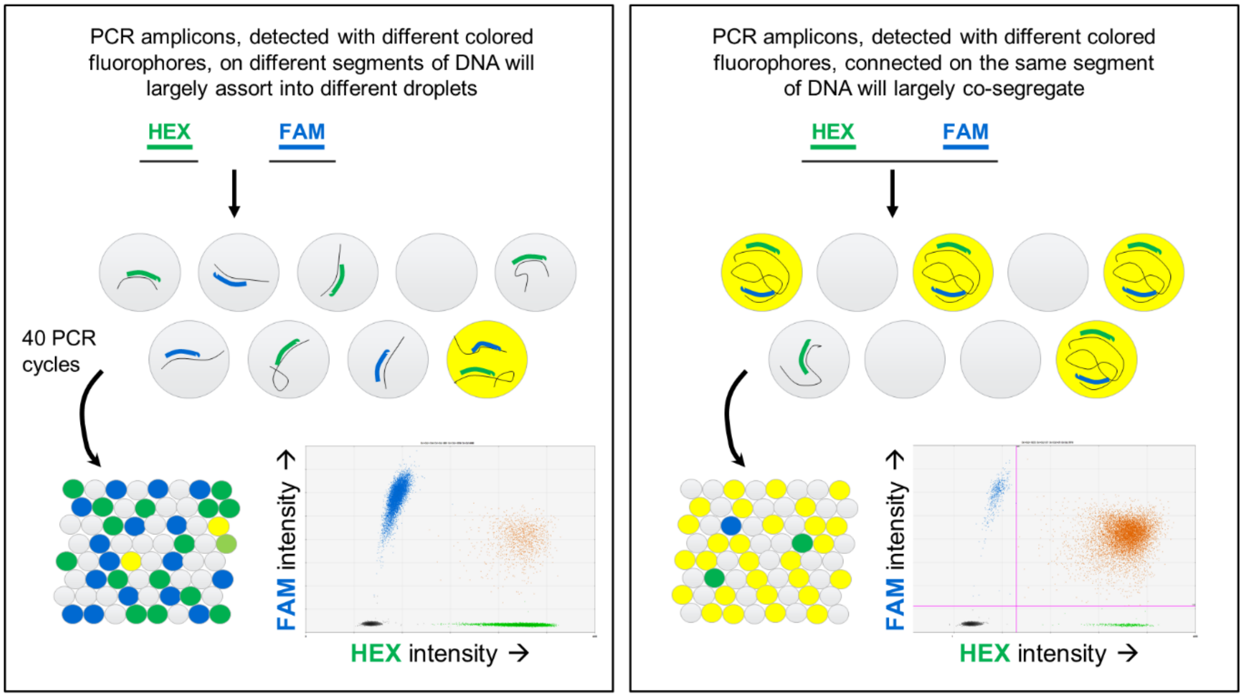

Supplementary Fig. 4 | Molecular analysis of hFVIII-SQ vector genomes for quantification of circular episomal forms of hFVIII-SQ in liver biopsy samples.

a) Drop-phase ddPCR provides information on contiguity of vector genomes. A combination of enzymatic treatment followed by ddPCR was used to detect episomes. ddPCR provides an absolute count of target DNA copies per input. The method partitions the DNA sample into 20,000 droplets by water-oil emulsion and PCR amplification is carried out within each droplet. If the target DNA concentration is within the dynamic range of the instrument, the droplets will either be read as positives or negatives which determine the absolute quantities of target DNA in the reaction. Reproduced from Sihn et al.[2] under Creative Commons license CC BY-NC-ND 4.0, ©2022 BioMarin Pharmaceutical Inc. Available at: https://www.cell.com/molecular-therapy-family/methods/fulltext/S2329-0501(21)00192-3 *(Supplemental Figure 4).*

FAM, HEX, fluorescent tags; hFVIII-SQ, PCR, polymerase chain reaction.

Continued

b

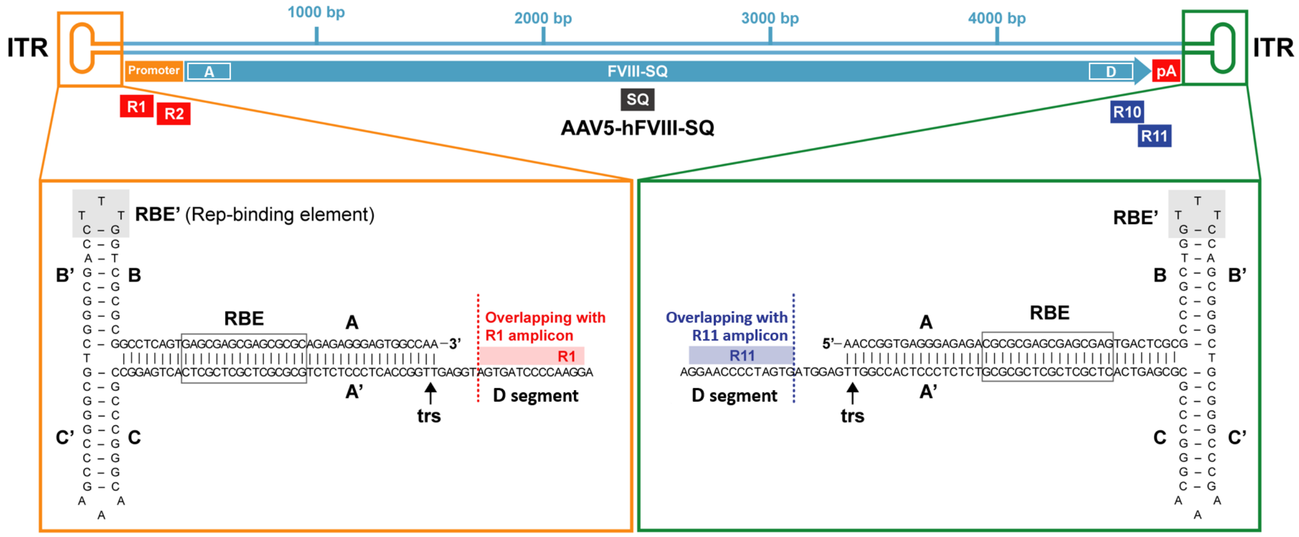

c

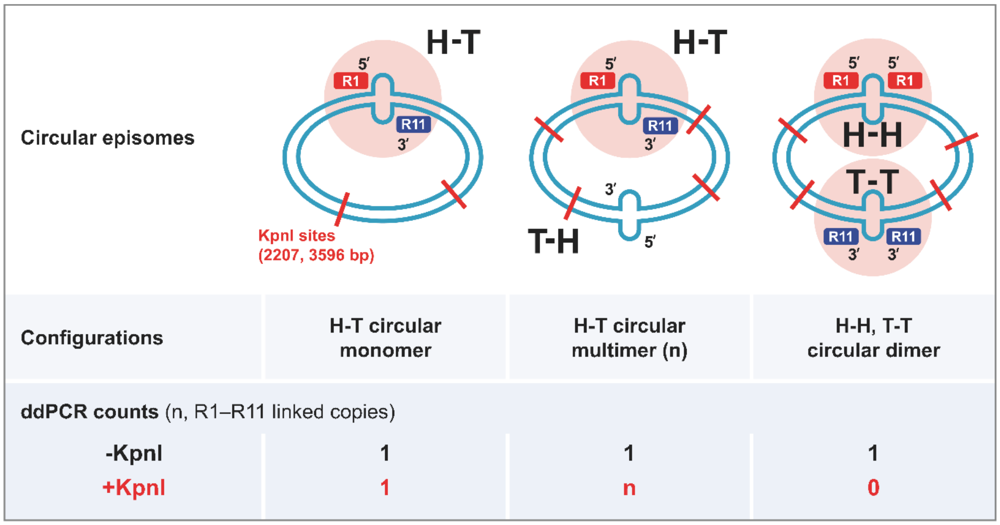

Supplementary Fig. 4 (continued)

b) Schematic of hFVIII-SQ vector genome with the locations of amplicons and primers/probes to detect vector genomes and ITR fusions. Full-length hFVIII-SQ vector genomes were measured by R1-R11 linked genomes in drop-phase ddPCR reactions. Forward primers in R1 or R11 amplicons overlap with segment D of ITRs; thus, droplets containing positive R1 and R11 amplicons indicate full-length genomes containing segment D of 5′ ITR, the promoter, hFVIII-SQ transgene, poly adenylation signal and segment D of 3′ ITR, presumably capable of giving rise to stable hFVIII-SQ transcription. Reproduced from Sihn et al.[2] under Creative Commons license CC BY-NC-ND 4.0, ©2022 BioMarin Pharmaceutical Inc. ITR structure originally adapted from Gonçalves.[18] Available at: https://www.cell.com/molecular-therapy-family/methods/fulltext/S2329-0501(21)00192-3 *(Supplemental Figure 2).*
c) Concatemers are separated into individual units following KpnI treatment and counted by ddPCR.

bp, base pairs; CDS, coding sequence; ddPCR, droplet digital polymerase chain reaction; hFVIII-SQ, B domain-deleted human factor VIII; H, head; ITR, inverted terminal repeat; pA, polyadenylation signal; RBE, Rep-binding element; T, tail; trs, terminal resolution site.

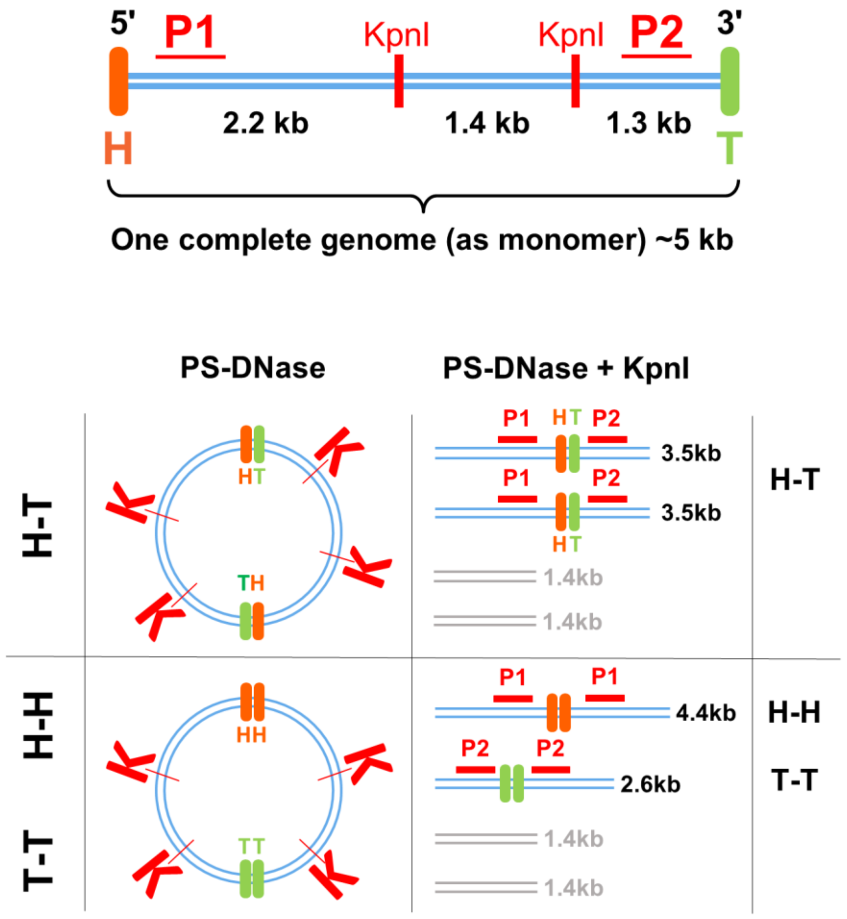

Supplementary Fig. 5 | Locations of the Southern Blot probes on various vector genome configurations and expected restriction-digested fragment sizes during Southern blotting.

Reproduced from Sihn et al.[2] under Creative Commons license CC BY-NC-ND 4.0, ©2022 BioMarin Pharmaceutical Inc. Available at: https://www.cell.com/molecular-therapy-family/methods/fulltext/S2329-0501(21)00192-3 *(Figure 4F)*

P1 and P2 are biotinylated probes used in Southern blotting. Sites marked with K are cut by KpnI.

H, head (5’ end); H-H, head-to-head orientation; H-T, head-to-tail orientation; kb, kilobases; P1, probe 1; P2, probe 2; PS-DNase, Plasmid Safe™ ATP-Dependent DNAase; T, tail (3’ end).

a

Annotation Cluster 1	Enrichment Score: 14.63	Count	P value	Benjamini
GOTERM_BP_DIRECT	translational initiation	34	1.3E-24	2.5E-21
GOTERM_BP_DIRECT	SRP-dependent cotranslational protein targeting to membrane	26	4.6E-20	4.3E-17
GOTERM_BP_DIRECT	viral transcription	27	3.4E-19	2.1E-16
UP_KEYWORDS	ribosomal protein	30	4.8E-18	5.7E-16
UP_KEYWORDS	ribonucleoprotein	36	1.6E-17	1.4E-15
GOTERM_BP_DIRECT	nuclear-transcribed mRNA catabolic process, nonsense-mediated decay	26	2.1E-17	1.0E-14
GOTERM_BP_DIRECT	rRNA processing	31	1.8E-15	6.7E-13
KEGG_PATHWAY	ribosome	29	2.0E-15	4.5E-13
GOTERM_BP_DIRECT	translation	32	2.6E-14	8.3E-12
GOTERM_MF_DIRECT	structural constituent of ribosome	30	4.0E-14	1.3E-11
GOTERM_CC_DIRECT	ribosome	25	2.0E-13	2.7E-11
GOTERM_CC_DIRECT	cytosolic large ribosomal subunit	15	1.8E-10	1.4E-8
GOTERM_CC_DIRECT	cytosolic small ribosomal subunit	11	6.6E-8	4.5E-6
GOTERM_CC_DIRECT	focal adhesion	27	4.8E-7	2.8E-5
**Annotation Cluster 2**	**Enrichment Score: 5.81**	**Count**	**P value**	**Benjamini**
GOTERM_MF_DIRECT	cadherin binding involved in cell-cell adhesion	23	1.1E-6	1.8E-4
GOTERM_BP_DIRECT	cell-cell adhesion	22	1.3E-6	2.7E-4
GOTERM_CC_DIRECT	cell-cell adherens junction	23	2.5E-6	9.2E-5
**Annotation Cluster 3**	**Enrichment Score: 5.17**	**Count**	**P value**	**Benjamini**
GOTERM_BP_DIRECT	formation of translation preinitiation complex	8	5.7E-7	1.5E-4
GOTERM_BP_DIRECT	regulation of translational initiation	9	1.2E-6	2.9E-4
UP_KEYWORDS	initiation factor	10	1.9E-6	7.5E-5
GOTERM_MF_DIRECT	translation initiation factor activity	10	1.0E-5	1.1E-3
KEGG_PATHWAY	RNA transport	19	1.1E-5	1.3E-3
GOTERM_CC_DIRECT	eukaryotic 43S preinitiation complex	6	1.2E-5	3.5E-4
GOTERM_CC_DIRECT	eukaryotic 48S preinitiation complex	6	1.2E-5	3.5E-4
GOTERM_CC_DIRECT	eukaryotic translation initiation factor 3 complex	6	2.4E-5	6.4E-4
UP_KEYWORDS	protein biosynthesis	13	5.1E-5	1.2E-3
**Annotation Cluster 4**	**Enrichment Score: 5.17**	**Count**	**P value**	**Benjamini**
GOTERM_MF_DIRECT	unfolded protein binding	14	1.5E-6	1.9E-4
UP_KEYWORDS	chaperone	17	2.7E-6	8.6E-5
GOTERM_BP_DIRECT	protein folding	15	7.4E-5	1.3E-2

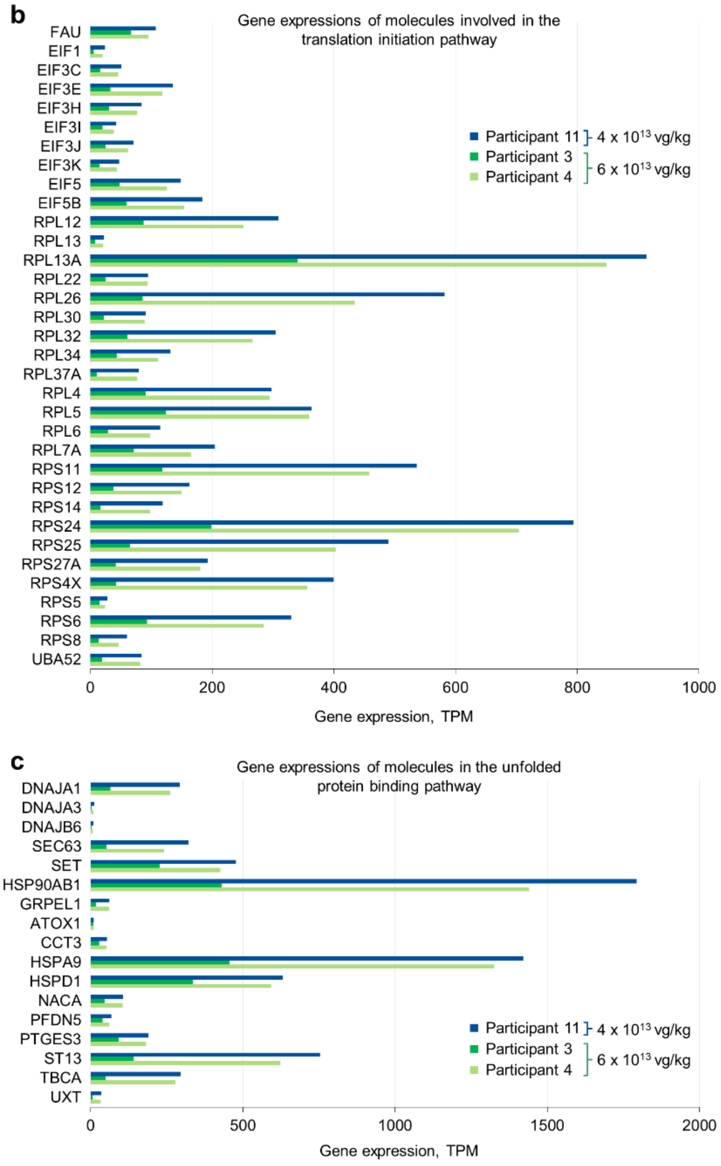

Supplementary Fig. 6 | Expression of molecules in the translation initiation and unfolded protein binding pathway positively correlated with FVIII activity. a) Gene ontology clustering of genes whose expression correlated positively with plasma FVIII activity ($r \geq 0.9$ and $p < 0.01$) using the Benjamini-Hochberg method for multiple hypothesis testing correction.[19] Pathway enrichment with expression of molecules positively correlated with plasma FVIII activity levels (using DAVID Functional Annotation Clustering. https://david.ncifcrf.gov/).[20,21] Gene expressions of molecules in **b)** the translation initiation pathway and **c)** unfolded protein binding pathway.

TPM, transcripts per million. *Gene names and descriptions: b. Molecules in the translation initiation pathway*. **FAU**, Finkel-Biskis-Reilly murine sarcoma virus (FBR-MuSV) ubiquitously expressed; **EIF1**, eukaryotic translation initiation factor 1; **EIF3C**, eukaryotic translation initiation factor 3, subunit C; **EIF3E**, eukaryotic translation initiation factor 3, subunit E; **EIF3H**, eukaryotic translation initiation factor 3, subunit H; **EIF3I**, eukaryotic translation initiation factor 3, subunit I; **EIF3J**, eukaryotic translation initiation factor 3, subunit J; **EIF3K**, eukaryotic translation initiation factor 3, subunit K; **EIF5**, eukaryotic translation initiation factor 5; **EIF5B**, eukaryotic translation initiation factor 5B; **RPL12**, ribosomal protein L12; **RPL13**, ribosomal protein L13; **RPL13A**, ribosomal protein L13a; **RPL22**, ribosomal protein L22; **RPL26**, ribosomal protein L26; **RPL30**, ribosomal protein L30; **RPL32**, ribosomal protein L32; **RPL34**, ribosomal protein L34; **RPL37A**, ribosomal protein L37a; **RPL4**, ribosomal protein L4; **RPL5**, ribosomal protein L5; **RPL6**, ribosomal protein L6; **RPL7A**, ribosomal protein L7a; **RPS11**, ribosomal protein S11; **RPS12**, ribosomal protein S12; **RPS14**, ribosomal protein S14; **RPS24**, ribosomal protein S24; **RPS25**, ribosomal protein S25; **RPS27A**, ribosomal protein S27a; **RPS4X**, ribosomal protein S4, X-linked; **RPS5**, ribosomal protein S5; **RPS6**, ribosomal protein S6; **RPS8**, ribosomal protein S8; **UBA52**, ubiquitin A-52 residue ribosomal protein fusion product 1. *c. Molecules in the unfolded protein binding pathway*. **DNAJA1**, DnaJ (Hsp40) homolog, subfamily A, member 1; **DNAJA3**, DnaJ (Hsp40) homolog, subfamily A, member 3; **DNAJB6**, DnaJ (Hsp40) homolog, subfamily B, member 6; **SEC63**, SEC63 homolog (*S. cerevisiae*); **SET**, SET nuclear oncogene; **HSP90AB1**, heat shock protein 90kDa alpha (cytosolic), class B member 1; **GRPEL1**, GRPE-like 1, mitochondrial (*E. coli*); **ATOX1**, antioxidant 1 copper chaperone; **CCT3**, chaperonin containing TCP1, subunit 3 (gamma); **HSPA9**, heat shock 70kDa protein 9 (mortalin); **HSPD1**, heat shock 60kDa protein 1 (chaperonin); **NACA**, nascent polypeptide-associated complex alpha subunit; **PFDN5**, prefoldin subunit 5; **PTGES3**, prostaglandin E synthase 3 (cytosolic); **ST13**, suppression of tumorigenicity 13 (colon carcinoma) (Hsp70 interacting protein); **TBCA**, tubulin folding cofactor A; **UXT**, ubiquitously-expressed, prefoldin-like chaperone.

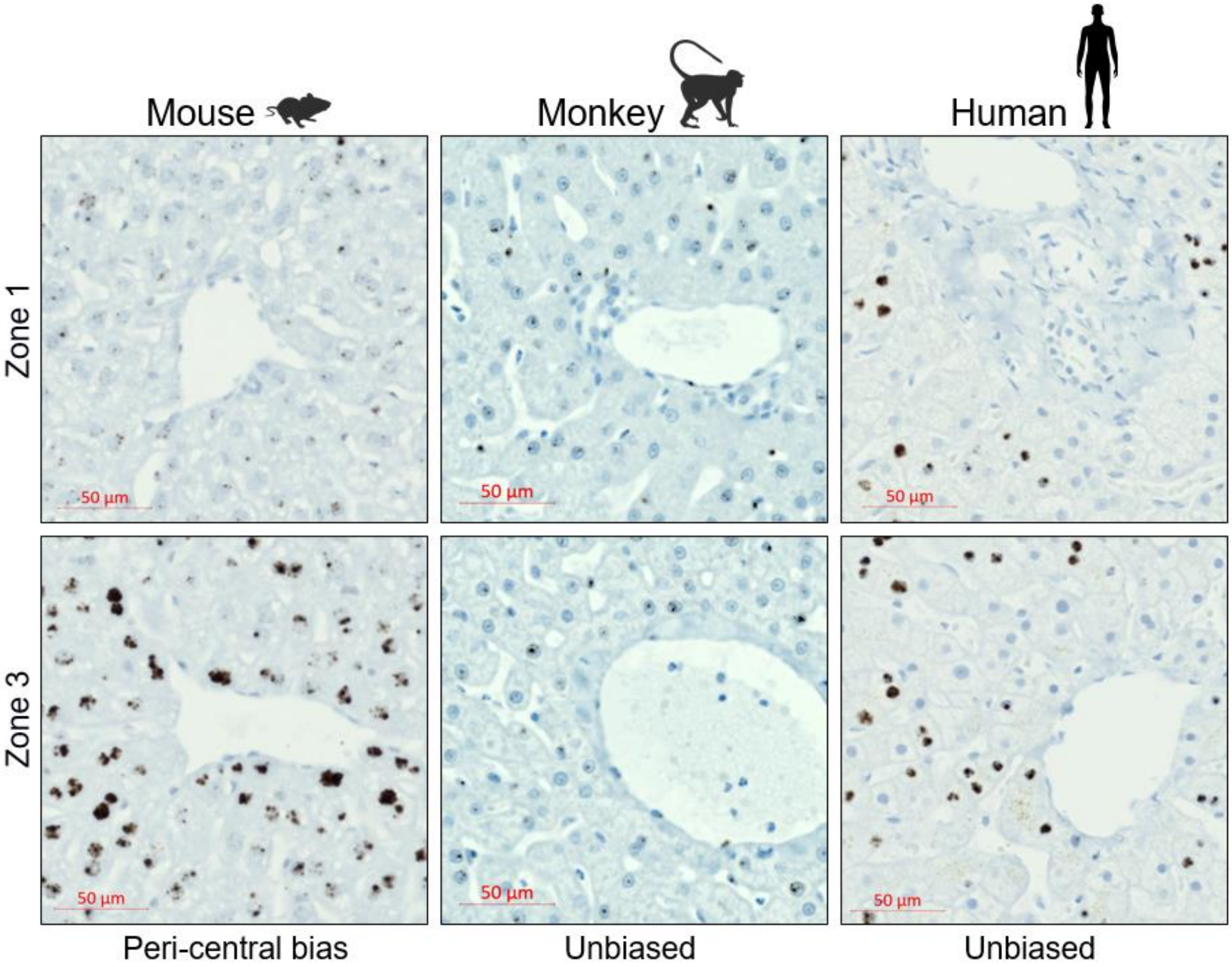

Supplementary Fig. 7 | Cross-species comparison of valoctocogene roxaparvovec transduction in hepatocytes. In situ hybridization analysis to detect vector genomes, showing peri-central distribution of hFVIII-SQ DNA (brown foci) in liver samples from Rag2-/- FVIII-/- double knockout mouse (5 weeks after vector administration) and unbiased distribution in healthy cynomolgus monkey (13 weeks) and human (Participant 3; 214 weeks) samples. Each focus (brown dot) represents at least one vector DNA molecule; it is possible to have multiple copies of vector genome within a single focus. Images were captured at 1600 × 1200 pixels and output at 300 ppi. Mouse and monkey liver samples were from animals in the preclinical studies described by Sihn et al.[2] Images from mouse are representative of 10 animals reviewed (two sections per animal). For monkey, images are representative of four individual animals (one liver section per animal). Additional images from the human biopsy study are shown in **Extended Data Fig. 2**, including zoom-out images that allow comparison of liver sections proximal and distal to central veins.

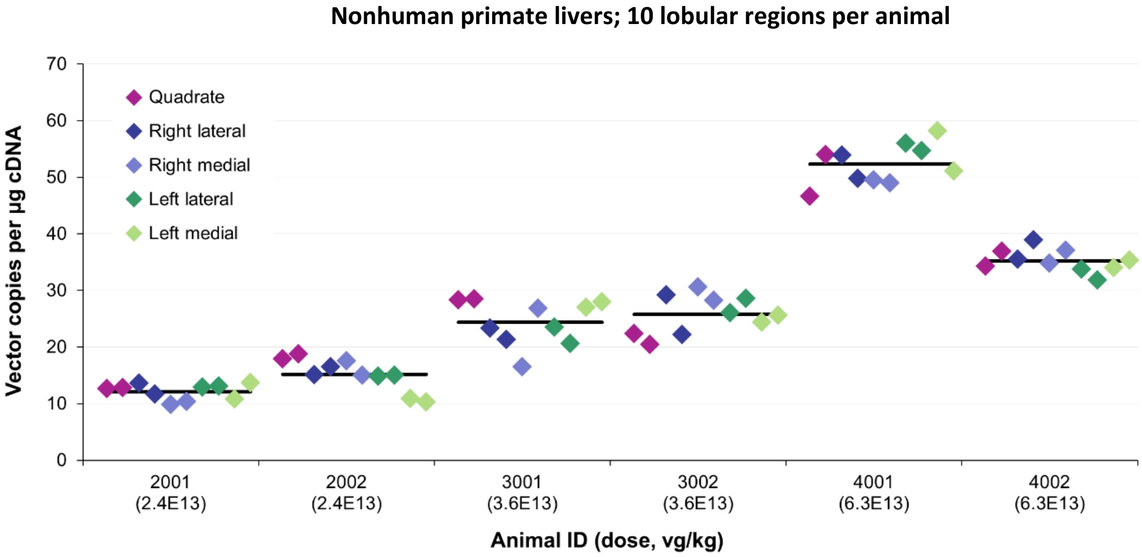

Supplementary Fig. 8 | Distribution of hFVIII-SQ vector genome in NHP livers after AAV5-FVIII-SQ gene transfer. Levels of hFVIII-SQ DNA detected by ddPCR[2] in liver samples from 10 different regions (two separate regions per lobe) in each of six cynomolgus monkeys dosed at 2.4×10^{13} vg/kg (n=2), 3.6×10^{13} vg/kg (n=2), and 6.3×10^{13} vg/kg (n=2), by methods described by Bunting et al.[3] Horizontal lines denote mean values for each set of 10 results.

vg, vector genomes.

References

1. Wang, D., Tai, P. W. L. & Gao, G. Adeno-associated virus vector as a platform for gene therapy delivery. *Nat. Rev. Drug Discov.* **18**, 358–378 (2019).
2. Sihn, C. R. et al. Molecular analysis of AAV5-hFVIII-SQ vector-genome-processing kinetics in transduced mouse and nonhuman primate livers. *Mol. Ther. Methods Clin. Dev.* **24**, 142–153 (2022).
3. Bunting, S. et al. Gene therapy with BMN 270 results in therapeutic levels of FVIII in mice and primates and normalization of bleeding in hemophilic mice. *Mol. Ther.* **26**, 496–509 (2018).
4. Wang, J. et al. Existence of transient functional double-stranded DNA intermediates during recombinant AAV transduction. *Proc. Natl Acad. Sci. USA* **104**, 13104–13109 (2007).
5. Vincent-Lacaze, N. et al. Structure of adeno-associated virus vector DNA following transduction of the skeletal muscle. *J. Virol.* **73**, 1949–1955 (1999).
6. Hirsch, M. L. et al. Oversized AAV transduction is mediated via a DNA-PKcs-independent, Rad51C-dependent repair pathway. *Mol. Ther.* **21**, 2205–2216 (2013).
7. Nakai, H., Storm, T. A. & Kay, M. A. Recruitment of single-stranded recombinant adeno-associated virus vector genomes and intermolecular recombination are responsible for stable transduction of liver in vivo. *J. Virol.* **74**, 9451–9463 (2000).
8. Duan, D. et al. Circular intermediates of recombinant adeno-associated virus have defined structural characteristics responsible for long-term episomal persistence in muscle tissue. *J. Virol.* **72**, 8568–8577 (1998).
9. Song, S., Laipis, P. J., Berns, K. I. & Flotte, T. R. Effect of DNA-dependent protein kinase on the molecular fate of the rAAV2 genome in skeletal muscle. *Proc. Natl Acad. Sci. USA* **98**, 4084–4088 (2001).
10. Nakai, H. et al. A limited number of transducible hepatocytes restricts a wide-range linear vector dose response in recombinant adeno-associated virus-mediated liver transduction. *J. Virol.* **76**, 11343–11349 (2002).
11. Nakai, H., Storm, T. A., Fuess, S. & Kay, M. A. Pathways of removal of free DNA vector ends in normal and DNA-PKcs-deficient SCID mouse hepatocytes transduced with rAAV vectors. *Hum. Gene Ther.* **14**, 871–881 (2003).
12. Penaud-Budloo, M. et al. Adeno-associated virus vector genomes persist as episomal chromatin in primate muscle. *J. Virol.* **82**, 7875–7885 (2008).
13. Yan, Z., Zak, R., Zhang, Y. & Engelhardt, J. F. Inverted terminal repeat sequences are important for intermolecular recombination and circularization of adeno-associated virus genomes. *J. Virol.* **79**, 364–379 (2005).
14. Song, S. et al. DNA-dependent PK inhibits adeno-associated virus DNA integration. *Proc. Natl Acad. Sci. USA* **101**, 2112–2116 (2004).
15. Gil-Farina, I. et al. Recombinant AAV integration is not associated with hepatic genotoxicity in nonhuman primates and patients. *Mol. Ther.* **24**, 1100–1105 (2016).
16. Nakai, H. et al. Extrachromosomal recombinant adeno-associated virus vector genomes are primarily responsible for stable liver transduction in vivo. *J. Virol.* **75**, 6969–6976 (2001).
17. Chandler, R. J. et al. Vector design influences hepatic genotoxicity after adeno-associated virus gene therapy. *J. Clin. Invest.* **125**, 870–880 (2015).
18. Gonçalves, M. A. Adeno-associated virus: from defective virus to effective vector. *Virol. J.* **2**, 43 (2005).
19. Benjamini, Y. & Hochberg, Y. Controlling the false discovery rate: a practical and powerful approach to multiple testing. *J. R. Stat. Soc. B* **57**, 289–300 (1995).
20. Huang, D. W., Sherman, B. T. & Lempicki, R. A. Systematic and integrative analysis of large gene lists using DAVID bioinformatics resources. *Nat. Protoc.* **4**, 44–57 (2009).
21. Huang, D. W., Sherman, B. T. & Lempicki, R. A. Bioinformatics enrichment tools: paths toward the comprehensive functional analysis of large gene lists. *Nucleic Acids Res.* **37**, 1–13 (2009).

Source data for Supplementary Figures

Supplementary Fig. 6 | Expression of molecules in the translation initiation and unfolded protein binding pathway positively correlated with FVIII activity.

b) Gene expressions of molecules in the translation initiation pathway, transcripts per million (TPM)

Geneid	Gene name	Gene description	P11	P4	P3	P15
ENSG00000149806	FAU	Finkel-Biskis-Reilly murine sarcoma virus (FBR-MuSV) ubiquitously expressed [Source:HGNC Symbol;Acc:3597]	107.6	95.3	66.9	77.8
ENSG00000173812	EIF1	eukaryotic translation initiation factor 1 [Source:HGNC Symbol;Acc:3249]	24.2	20.1	5.7	43.5
ENSG00000184110	EIF3C	eukaryotic translation initiation factor 3, subunit C [Source:HGNC Symbol;Acc:3279]	50.6	45.7	15.8	35
ENSG00000104408	EIF3E	eukaryotic translation initiation factor 3, subunit E [Source:HGNC Symbol;Acc:3277]	135.6	118.3	32.8	169
ENSG00000147677	EIF3H	eukaryotic translation initiation factor 3, subunit H [Source:HGNC Symbol;Acc:3273]	84	77	30.5	124.8
ENSG00000084623	EIF3I	eukaryotic translation initiation factor 3, subunit I [Source:HGNC Symbol;Acc:3272]	42.4	38.3	20.5	65
ENSG00000104131	EIF3J	eukaryotic translation initiation factor 3, subunit J [Source:HGNC Symbol;Acc:3270]	70.5	62.2	25.1	54.4
ENSG00000178982	EIF3K	eukaryotic translation initiation factor 3, subunit K [Source:HGNC Symbol;Acc:24656]	47.7	43.4	15	31
ENSG00000100664	EIF5	eukaryotic translation initiation factor 5 [Source:HGNC Symbol;Acc:3299]	148.7	126	48.2	116.5
ENSG00000158417	EIF5B	eukaryotic translation initiation factor 5B [Source:HGNC Symbol;Acc:30793]	184	154.3	59.8	120.9
ENSG00000197958	RPL12	ribosomal protein L12 [Source:HGNC Symbol;Acc:10302]	309.2	251.4	87.3	355
ENSG00000167526	RPL13	ribosomal protein L13 [Source:HGNC Symbol;Acc:10303]	22.8	20.8	8.3	28.9
ENSG00000142541	RPL13A	ribosomal protein L13a [Source:HGNC Symbol;Acc:10304]	913.5	847.7	340.5	843.8
ENSG00000116251	RPL22	ribosomal protein L22 [Source:HGNC Symbol;Acc:10315]	94.5	94.3	25.2	161.7
ENSG00000161970	RPL26	ribosomal protein L26 [Source:HGNC Symbol;Acc:10327]	581.8	434	85.9	619.4
ENSG00000156482	RPL30	ribosomal protein L30 [Source:HGNC Symbol;Acc:10333]	90.9	89.2	22.7	146
ENSG00000144713	RPL32	ribosomal protein L32 [Source:HGNC Symbol;Acc:10336]	304.2	266.5	60.8	455.9
ENSG00000109475	RPL34	ribosomal protein L34 [Source:HGNC Symbol;Acc:10340]	132	110.9	43.8	195.5
ENSG00000197756	RPL37A	ribosomal protein L37a [Source:HGNC Symbol;Acc:10348]	79.9	76.8	10.8	102.7
ENSG00000174444	RPL4	ribosomal protein L4 [Source:HGNC Symbol;Acc:10353]	297.8	295	90.8	314.8
ENSG00000122406	RPL5	ribosomal protein L5 [Source:HGNC Symbol;Acc:10360]	363.8	359.8	124.6	581.7
ENSG00000089009	RPL6	ribosomal protein L6 [Source:HGNC Symbol;Acc:10362]	114.7	97.6	28.6	137.5
ENSG00000148303	RPL7A	ribosomal protein L7a [Source:HGNC Symbol;Acc:10364]	204.8	165.8	71.3	315.5
ENSG00000142534	RPS11	ribosomal protein S11 [Source:HGNC Symbol;Acc:10384]	536	458.5	118.2	921.4
ENSG00000112306	RPS12	ribosomal protein S12 [Source:HGNC Symbol;Acc:10385]	163	150	37.4	284.9
ENSG00000164587	RPS14	ribosomal protein S14 [Source:HGNC Symbol;Acc:10387]	118.7	97.9	17	53.7
ENSG00000138326	RPS24	ribosomal protein S24 [Source:HGNC Symbol;Acc:10411]	793.7	704	198.8	865.5
ENSG00000118181	RPS25	ribosomal protein S25 [Source:HGNC Symbol;Acc:10413]	489.9	403.2	65.2	435.9
ENSG00000143947	RPS27A	ribosomal protein S27a [Source:HGNC Symbol;Acc:10417]	192.9	180.9	42.1	310.1
ENSG00000198034	RPS4X	ribosomal protein S4, X-linked [Source:HGNC Symbol;Acc:10424]	399.6	356.1	42.7	489.1
ENSG00000083845	RPS5	ribosomal protein S5 [Source:HGNC Symbol;Acc:10426]	27.8	24.1	15.4	47.8
ENSG00000137154	RPS6	ribosomal protein S6 [Source:HGNC Symbol;Acc:10429]	330	285.3	93.5	335.2
ENSG00000142937	RPS8	ribosomal protein S8 [Source:HGNC Symbol;Acc:10441]	59.9	46.4	13.5	76.2
ENSG00000221983	UBA52	ubiquitin A-52 residue ribosomal protein fusion product 1 [Source:HGNC Symbol;Acc:12458]	84.2	82.2	19	119.1

c) Gene expressions of molecules in the unfolded protein binding pathway, transcripts per million (TPM)

Geneid	Gene name	Gene description	P11	P4	P3	P15
ENSG00000086061	DNAJA1	DnaJ (Hsp40) homolog, subfamily A, member 1 [Source:HGNC Symbol;Acc:5229]	292.8	261.3	65.6	326.4
ENSG00000103423	DNAJA3	DnaJ (Hsp40) homolog, subfamily A, member 3 [Source:HGNC Symbol;Acc:11808]	11.3	9.3	4.6	19.4
ENSG00000105993	DNAJB6	DnaJ (Hsp40) homolog, subfamily B, member 6 [Source:HGNC Symbol;Acc:14888]	8.9	7.9	4.2	6.5
ENSG00000025796	SEC63	SEC63 homolog (S. cerevisiae) [Source:HGNC Symbol;Acc:21082]	321.8	240.7	52.8	291.6
ENSG00000119335	SET	SET nuclear oncogene [Source:HGNC Symbol;Acc:10760]	476.7	425.4	226.7	170.7
ENSG00000096384	HSP90AB1	heat shock protein 90kDa alpha (cytosolic), class B member 1 [Source:HGNC Symbol;Acc:5258]	1793.4	1440.5	430.8	849.8
ENSG00000109519	GRPEL1	GrpE-like 1, mitochondrial (E. coli) [Source:HGNC Symbol;Acc:19696]	62.2	59.4	18	46.9
ENSG00000177556	ATOX1	antioxidant 1 copper chaperone [Source:HGNC Symbol;Acc:798]	11	10.3	8.3	8.9
ENSG00000163468	CCT3	chaperonin containing TCP1, subunit 3 (gamma) [Source:HGNC Symbol;Acc:1616]	53.7	51.4	27.9	81.2
ENSG00000113013	HSPA9	heat shock 70kDa protein 9 (mortalin) [Source:HGNC Symbol;Acc:5244]	1421.6	1325.2	456.7	1615.5
ENSG00000144381	HSPD1	heat shock 60kDa protein 1 (chaperonin) [Source:HGNC Symbol;Acc:5261]	630.9	593.3	335.5	864.6
ENSG00000196531	NACA	nascent polypeptide-associated complex alpha subunit [Source:HGNC Symbol;Acc:7629]	107.3	104.3	46.3	144
ENSG00000123349	PFDN5	prefoldin subunit 5 [Source:HGNC Symbol;Acc:8869]	67.9	62.1	40	115.9
ENSG00000110958	PTGES3	prostaglandin E synthase 3 (cytosolic) [Source:HGNC Symbol;Acc:16049]	189.7	182.1	92.9	127.5
ENSG00000100380	ST13	suppression of tumorigenicity 13 (colon carcinoma) (Hsp70 interacting protein) [Source:HGNC Symbol;Acc:11343]	752.7	622.9	141.5	636.5
ENSG00000171530	TBCA	tubulin folding cofactor A [Source:HGNC Symbol;Acc:11579]	295.3	277.9	48.3	285.6
ENSG00000126756	UXT	ubiquitously-expressed, prefoldin-like chaperone [Source:HGNC Symbol;Acc:12641]	34.4	32.7	6.7	44.3

Supplementary Fig. 8 | Distribution of hFVIII-SQ vector genome in NHP livers after AAV5-FVIII-SQ gene transfer.

Levels of hFVIII-SQ DNA detected by ddPCR[17] in liver samples from 10 different regions (2 separate regions per lobe) in each of 6 cynomolgus monkeys

Animal ID (dose, vg/kg)	Result: Copies per µg cDNA	Mean
2001 (2.4E13) (N=10)	12.60	12.1
	12.80	
	13.60	
	11.70	
	9.87	
	10.40	
	12.90	
	13.10	
	10.80	
	13.70	
2002 (2.4E13) (N=10)	17.90	15.2
	18.80	
	15.10	
	16.50	
	17.50	
	15.00	
	14.80	
	15.00	
	10.90	
	10.30	
3001 (3.6E13) (N=10)	28.30	24.4
	28.50	
	23.30	
	21.30	
	16.50	
	26.80	
	23.50	
	20.60	
	27.00	
	28.00	

Animal ID (dose, vg/kg)	Result: Copies per µg cDNA	Mean
3002 (3.6E13) (N=10)	22.40	25.8
	20.40	
	29.20	
	22.20	
	30.60	
	28.20	
	26.00	
	28.60	
	24.40	
	25.60	
4001 (6.3E13) (N=10)	46.60	52.3
	54.00	
	53.90	
	49.80	
	49.50	
	49.00	
	56.00	
	54.70	
	58.20	
	51.10	
4002 (6.3E13) (N=10)	34.30	35.2
	36.90	
	35.50	
	38.90	
	34.80	
	37.10	
	33.70	
	31.80	
	34.00	
	35.30	
