# Supplementary material for: Interindividual variability in transgene mRNA and protein production following adeno-associated virus gene therapy for hemophilia A
Source: Nat Med. 2022 Apr 11;28(4):789–97. doi: 10.1038/s41591-022-01751-0 (PMC9018415; doi:10.1038/s41591-022-01751-0)
Supplement: Supplementary file 2 — Reporting Summary [file 41591_2022_1751_MOESM2_ESM.pdf]

nature research

Corresponding author(s): Sylvia Fong

Last updated by author(s): Jan 31, 2022

Reporting Summary

Nature Research wishes to improve the reproducibility of the work that we publish. This form provides structure for consistency and transparency in reporting. For further information on Nature Research policies, see our Editorial Policies and the Editorial Policy Checklist.

Statistics

For all statistical analyses, confirm that the following items are present in the figure legend, table legend, main text, or Methods section.

n/a	Confirmed	
☐	☒	The exact sample size (n) for each experimental group/condition, given as a discrete number and unit of measurement
☐	☒	A statement on whether measurements were taken from distinct samples or whether the same sample was measured repeatedly
☐	☒	The statistical test(s) used AND whether they are one- or two-sided<br>*Only common tests should be described solely by name; describe more complex techniques in the Methods section.*
☐	☒	A description of all covariates tested
☐	☒	A description of any assumptions or corrections, such as tests of normality and adjustment for multiple comparisons
☐	☒	A full description of the statistical parameters including central tendency (e.g. means) or other basic estimates (e.g. regression coefficient) AND variation (e.g. standard deviation) or associated estimates of uncertainty (e.g. confidence intervals)
☐	☒	For null hypothesis testing, the test statistic (e.g. F, t, r) with confidence intervals, effect sizes, degrees of freedom and P value noted<br>*Give P values as exact values whenever suitable.*
☒	☐	For Bayesian analysis, information on the choice of priors and Markov chain Monte Carlo settings
☒	☐	For hierarchical and complex designs, identification of the appropriate level for tests and full reporting of outcomes
☐	☒	Estimates of effect sizes (e.g. Cohen's d, Pearson's r), indicating how they were calculated

Our web collection on statistics for biologists contains articles on many of the points above.

Software and code

Policy information about availability of computer code

Data collection	Liver sections for histological evaluation were imaged and analyzed on QuPath version 0.2.3.<br>In droplet digital polymerase chain reaction (ddPCR) analyses, samples were read using a QX200 droplet reader (Bio-Rad, Hercules, CA) and quantified using QuantaSoft™ software, version 1.7.4.0917 (Bio-Rad, Hercules, CA).
Data analysis	Numerical and statistical analyses were performed using GraphPad Prism 7.01 (GraphPad Software).<br>NA sequencing analyses were performed using ROSALIND® version 3.19.0.5 (https://rosalind.onramp.bio/), with a HyperScale architecture developed by ROSALIND, Inc. (San Diego, CA).<br>Image analysis was performed using Visiopharm version 2020.09 (Hoersholm, Denmark).<br>Several database sources were referenced for pathway enrichment analysis, including Interpro (PANTHER version 15.0, http://www.pantherdb.org/), NCBI (https://www.ncbi.nlm.nih.gov/nuccore/), MSigDB (Molecular Signature Database) version 7.2 (http://www.gsea-msigdb.org/gsea/index.jsp), REACTOME (Reactome database release 73, https://reactome.org/), WikiPathways (https://www.wikipathways.org), and DAVID (The Database for Annotation, Visualization and Integrated Discovery) version 6.8 (https://david.ncifcrf.gov/).

For manuscripts utilizing custom algorithms or software that are central to the research but not yet described in published literature, software must be made available to editors and reviewers. We strongly encourage code deposition in a community repository (e.g. GitHub). See the Nature Research guidelines for submitting code & software for further information.

Data

Policy information about availability of data

All manuscripts must include a data availability statement. This statement should provide the following information, where applicable:

- Accession codes, unique identifiers, or web links for publicly available datasets
- A list of figures that have associated raw data
- A description of any restrictions on data availability

Due to the very small number of participants in this study, drawn from the limited number of individuals in this rare disease population, the gene expression profiles/sequencing libraries generated for each participant have not been shared via a public repository to avoid potentially compromising patients' identities. However, the de-identified individual participant data that underlie the results reported in this article (including text, tables, figures, and appendices) will be made available together with the research protocol and data dictionaries, for noncommercial, academic purposes. Additional supporting documents may be available upon request.

Investigators will be able to request access to these data and supporting documents via a website (www.BioMarin.com) beginning 6 months and ending 2 years after publication. Data associated with any ongoing development program will be made available within 6 months after approval of relevant product.
Requests must include a research proposal clarifying how the data will be used, including proposed analysis methodology. Research proposals will be evaluated relative to publicly available criteria available at www.BioMarin.com to determine if access will be given, contingent upon execution of a data access agreement with BioMarin Pharmaceutical Inc.

Field-specific reporting

Please select the one below that is the best fit for your research. If you are not sure, read the appropriate sections before making your selection.

☒ Life sciences ☐ Behavioural & social sciences ☐ Ecological, evolutionary & environmental sciences

For a reference copy of the document with all sections, see nature.com/documents/nr-reporting-summary-flat.pdf

Life sciences study design

All studies must disclose on these points even when the disclosure is negative.

Sample size	Substudy of 5 participants dosed with valoctocogene roxaparvovec treated in a phase 1/2 clinical trial. No sample size calculation was performed; this was an exploratory substudy and sample numbers were limited by the invasive nature of the liver biopsy procedure and by the small clinical trial population. Of the 15 men who enrolled and were dosed in the phase 1/2 clinical trial, all were invited to take part in this liver biopsy substudy, and 5 consented to participate.
Data exclusions	There were no data exclusions among the 5 participants who underwent biopsy.
Replication	For each substudy participant, multiple sections for histopathological and molecular analyses were derived from a single biopsy specimen. For molecular analyses involving ddPCR, at least 3 technical replicates were used per sample, per condition. All attempts at replication were successful.
Randomization	This was a planned substudy of a non-randomized, open-label, phase 1/2, dose-escalation, safety, tolerability and efficacy clinical study in which all participants received a single intravenous dose of valoctocogene roxaparvovec. There was no randomization to study interventions in either the clinical study or the liver biopsy substudy. Three naïve human liver samples were used as negative controls for molecular analyses in the liver biopsy study.
Blinding	Histopathological review of liver biopsy specimens was performed by a central expert pathologist blinded to subject characteristics and clinical information. For all other analyses, the analysts (BY, CRS, CR, SL) were blinded to group allocations and to the subject-associated information during data collection. Unblinding of the data findings and retrieval of corresponding subjects' plasma FVIII levels for subsequent evaluation was performed by SF.

Reporting for specific materials, systems and methods

We require information from authors about some types of materials, experimental systems and methods used in many studies. Here, indicate whether each material, system or method listed is relevant to your study. If you are not sure if a list item applies to your research, read the appropriate section before selecting a response.

Materials & experimental systems

n/a	Involved in the study	
☐	☒	Antibodies
☒	☐	Eukaryotic cell lines
☒	☐	Palaeontology and archaeology
☐	☒	Animals and other organisms
☐	☒	Human research participants
☐	☒	Clinical data
☒	☐	Dual use research of concern

Methods

n/a	Involved in the study	
☒	☐	ChIP-seq
☒	☐	Flow cytometry
☒	☐	MRI-based neuroimaging

Antibodies

Antibodies used

Anti-FVIII antibody Abcam (1:500, Cambridge, MA, cat. #ab139391), anti-GRP78 antibody (1:1000, Cell Signaling Technology, MA, cat. C50B12), anti-LAMP2 (1:100, Abcam, MA, cat. ab25631) diluted in Ventana Reaction Buffer (Ventana Medical Systems, AZ, USA; cat. # 950-300).

Secondary antibodies were donkey anti-sheep IgG (H+L) cross-adsorbed secondary antibody conjugated to Alexa Fluor 647 (1:1000, A-21448; Thermo Fisher Scientific, MA); donkey anti-rabbit IgG (H+L) highly cross-adsorbed secondary antibody conjugated with Alexa Fluor 555 (1:1000, A-21206; Thermo Fisher Scientific, MA); donkey anti-mouse IgG (H+L) cross-adsorbed secondary antibody conjugated to Alexa Fluor 488 (1:500, A-31570, Thermo Fisher Scientific, MA).

Validation

The anti-FVIII antibody was validated to detect human FVIII-SQ protein using murine liver tissues from mice treated with either vehicle or AAV5-hFVIII-SQ. Specific hFVIII immunostaining was detected in hepatocytes, but not endothelial cells. No immunoreactivity was detected in the vehicle treated animals. Further confirmation of antibody specificity was evaluated using human FFPE liver tissues. Immunoreactivity was localized to endothelial cells within the liver tissue, where endogenous FVIII is produced.

The anti-GRP78 antibody specificity was confirmed using HepG2 cells treated with and without thapsigargin, a compound known to upregulate the expression of GRP78. In addition, using human FFPE liver tissue, GRP78 and Calreticulin, another ER protein, were shown to give similar staining patterns.
References:
https://www.cellsignal.com/products/primary-antibodies/bip-c50b12-rabbit-mab/3177.
Li WW, Alexandre S, Cao X, Lee AS. Transactivation of the grp78 promoter by Ca2+ depletion. A comparative analysis with A23187 and the endoplasmic reticulum Ca(2+)-ATPase inhibitor thapsigargin. J Biol Chem 1993;268:12003-9.

The anti-LAMP2 antibody was thoroughly characterized by Abcam to specifically bind human LAMP2 protein using Flow Cytometry, western blot and immunohistochemistry. Subcellular localization to lysosomes was confirmed in human FFPE liver tissues.
Reference:
https://www.abcam.com/lamp2-antibody-h4b4-lysosome-marker-ab25631.html.

Animals and other organisms

Policy information about studies involving animals; ARRIVE guidelines recommended for reporting animal research

Laboratory animals

Male Rag2-/- FVIII-/- double knockout mice, an immune-compromised hemophilia A model that recapitulates the bleeding phenotype of human hemophilia A and minimizes the chance of antibody production against foreign protein; animals were 8–9 weeks old at the time of dosing.

Healthy male cynomolgus monkeys screened negative for total antibodies and neutralizing factors against AAV5 were 3–4 years old and weighed between 2.5 and 3.5 kg at the time of dosing.

Wild animals

The study did not involve wild animals.

Field-collected samples

The study did not involve samples collected from the field.

Ethics oversight

All in vivo animal procedures were performed in accordance with institutional guidelines under protocols approved by the Institutional Animal Care and Use Committees of the Buck Institute, Novato, CA (mice) and the Charles River Laboratories facility, Reno, NV (monkeys).

Note that full information on the approval of the study protocol must also be provided in the manuscript.

Human research participants

Policy information about studies involving human research participants

Population characteristics

Five men aged 23-37 years with severe hemophilia A. Participants had received gene replacement therapy with a single infusion of valoctocogene roxaparvovec (AAV5-hFVIII-SQ) at doses of 6e12, 4e13, or 6e13 vector genomes (vg) per kg, 2.6 to 4.1 years prior to undergoing transjugular or percutaneous liver biopsy for the current substudy.

Recruitment	Of the 15 men who enrolled in the phase 1/2 clinical trial, all were invited to take part in this liver biopsy substudy, and five consented to participate.
Ethics oversight	The protocol of the phase 1/2 valoctocogene roxaparvovec (AAV5-hFVIII-SQ) dose escalation, safety, and efficacy study (NCT02576795) was approved by South Central – Oxford A Research Ethics Committee, Bristol Research Ethics Committee Centre, Bristol BS1 2NT, United Kingdom, and the study was carried out in accordance with relevant national regulations, and the International Committee for Harmonisation Guidelines for Good Clinical Practice, and the principles of the Declaration of Helsinki. All participants provided written informed consent. Separate consent was sought for participation in the liver biopsy substudy. Participants received compensation for pre-biopsy and biopsy visits in the form of reimbursement to cover their expenses and time.<br><br>Normal liver samples from healthy donors were sourced from the following commercial providers of de-identified human biospecimens. Each provider confirmed that tissues had been collected with informed consent for their use for research purposes: AMSBio LLC, Cambridge, MA; BioIVT, Westbury, NY; Cureline, South San Francisco, CA; Discovery Life Sciences LLC, Huntsville, AL; Dx BioSamples, San Diego, CA; iSPecimen, Lexington, MA; and US Biolab, Rockville, MD.

Note that full information on the approval of the study protocol must also be provided in the manuscript.

Clinical data

Policy information about clinical studies

All manuscripts should comply with the ICMJE guidelines for publication of clinical research and a completed CONSORT checklist must be included with all submissions.

Clinical trial registration	ClinicalTrials.gov number, NCT02576795; EudraCT number, 2014-003880-38
Study protocol	The full clinical trial protocol is available at https://www.nejm.org/doi/suppl/10.1056/NEJMoa1908490/suppl_file/nejmoa1908490_protocol.pdf<br><br>This optional liver biopsy substudy was added to the protocol and is included in Amendment 8.
Data collection	Participants were enrolled into the liver biopsy substudy between July 2019 and February 2020 at three hemophilia centers in the UK. Participants underwent a transjugular or ultrasound-guided percutaneous liver biopsy performed according to the standard procedures at their institution. Frozen samples were shipped to the sponsor on dry ice and stored at −80°C, and formalin-fixed paraffin-embedded (FFPE) samples were shipped and stored at ambient temperature.<br><br>Gross pathology and histopathology were evaluated by pathologists at the local sites and centrally by an independent expert liver pathologist at the Department of Pathology, University of California San Francisco, San Francisco, CA, USA. Molecular analyses were performed at BioMarin Pharmaceutical Inc., Novato, CA, USA.
Outcomes	Outcomes of this exploratory substudy were measured in liver biopsy samples obtained from five participants 2.6–4.1 years after gene transfer with valoctocogene roxaparvovec:<br><br>Liver histopathology and safety evaluations of liver biopsy FFPE samples from each participant were reported descriptively with reference to published scoring/staging systems, to identify any adverse findings related to steatosis, inflammation, fibrosis, and overall liver architecture; when available, on-slide controls were reviewed for comparison.<br><br>The transduction pattern of AAV5-hFVIII-SQ was evaluated by observation of hepatocellular distribution of hFVIII-SQ DNA in FFPE liver biopsy sections, determined by in situ hybridization (ISH).<br><br>The extent of transduction was determined by measuring the percentage of hepatocytes transduced with hFVIII-SQ genomes using ISH followed by quantitative image analysis (percentage of hepatocyte nuclei stained positive for hFVIII-SQ DNA – mean counts across 10 images that spanned ≥50% of the biopsy).<br><br>Characterization and quantification of circular and various other AAV-hFVIII-SQ episomal vector genome forms was determined by quantitative, drop-phase droplet digital PCR (ddPCR) and by qualitative Southern blotting analyses performed on DNA isolated from biopsy samples that were treated with various DNA digestion enzymes and using custom-generated primers/probe sets.<br><br>Transgene expression was measured by quantification of hFVIII-SQ vector RNA transcripts in liver biopsies using reverse transcription following by ddPCR and normalized to 3 different endogenous reference RNAs.<br><br>Hepatic expression of valoctocogene roxaparvovec-derived hFVIII-SQ protein was determined by immunohistochemical (IHC) analysis of hFVIII co-localizing in lysosomes or endoplasmic reticulum (ER) of the hepatocytes, using confocal microscopy with LAMP2 and GRP78 as organelle-specific markers for lysosomes and ER, respectively. Hepatocytes were scored as either positive or negative for hFVIII protein. Initial methods to detect hFVIII-SQ protein expression and distribution in hepatocytes using IHC followed by epifluorescence microscopy were unsuccessful due to high background levels from recombinant hFVIII administered prior to the biopsy procedure to minimize bleeding risk.<br><br>Additional exploratory analyses were performed to investigate molecular mechanisms mediating variability in hFVIII-SQ RNA levels and determine covariate gene expression with hFVIII-SQ RNA and protein expression.
