# Supplementary material for: Interindividual variability in transgene mRNA and protein production following adeno-associated virus gene therapy for hemophilia A
Source: Nat Med. 2022 Apr 11;28(4):789–97. doi: 10.1038/s41591-022-01751-0 (PMC9018415; doi:10.1038/s41591-022-01751-0)

Source data, Figure 1c

Hepatocyte nuclei positive for hFVIII-SQ DNA by ISH

Percent positive nuclei: mean counts across 11 (Participants 1 and 11) or 27–28 (Participants 15, 3 and 4) images per biopsy

Participant 1<br>6e12 vg/kg	Participant 11<br>4e13 vg/kg	Participant 15<br>4e13 vg/kg	Participant 3<br>6e13 vg/kg	Participant 4<br>6e13 vg/kg
3.2	22.7	9.64	44.09	51.72
1.9	26.3	4.13	42.76	52.88
2.2	38.7	33.62	34.81	62.07
1.1	41.1	28.33	38.06	74.19
1.1	27.4	31.97	44.63	52.83
0.3	27.4	52.54	37.69	80.81
1	18.5	39.13	47.06	53.97
1.6	36.5	36.36	34.19	41.82
1.2	36.5	33.88	71.43	43.4
0	41.2	31.9	52.03	52.5
0.4	36	42.86	33.04	52.76
		53.1	39.47	49.57
		18.49	19.01	52.81
		46.03	34.55	69.23
		34.26	43.66	51.79
		40	34.21	43.14
		41.12	69.37	31.96
		30.28	48.25	57.14
		25.85	53.33	38.6
		12.8	42.11	36.75
		36.45	52.69	51.08
		34.48	28.57	47.54
		27.03	72.28	67.14
		31.36	48.54	30.93
		15.32	50.44	48.67
		22.95	46.96	52.8
		46.85	62.75	44.35
			38.79	63

Source data, Figure 1d

Circular genomes by drop-phase ddPCR

Circular genomes (full-length or H-T ITR-fused) detected in patient liver biopsy samples via drop-phase ddPCR following DNA sample treatment with PS-DNase and KpnI (mean copies per diploid genome)

	Full-Length	ITR-fused
Participant 1, 6e12 vg/kg	0.10	0.1
Participant 11, 4e13 vg/kg	1.66	1.9
Participant 15, 4e13 vg/kg	1.29	1.6
Participant 3, 6e13 vg/kg	3.11	3.7
Participant 4, 6e13 vg/kg	4.24	5.2

PS-DNase+KpnI
(total circular vg, H-T only)

Source data, Figure 1e

Southern blotting, uncropped images used in Fig. 1e and Extended Data Fig. 3f

bp, basepairs; d, dimer; H-H, head-to-head orientation; H-T, head-to-tail orientation; kbp, kilobase pairs; LM, linear markers; m, monomer; PS+, DNA samples treated with PS-DNase; PS+ KpnI+, DNA samples treated with PS-DNase followed by KpnI restriction enzyme digest; SC, supercoiled markers; T-T, tail-to-tail orientation

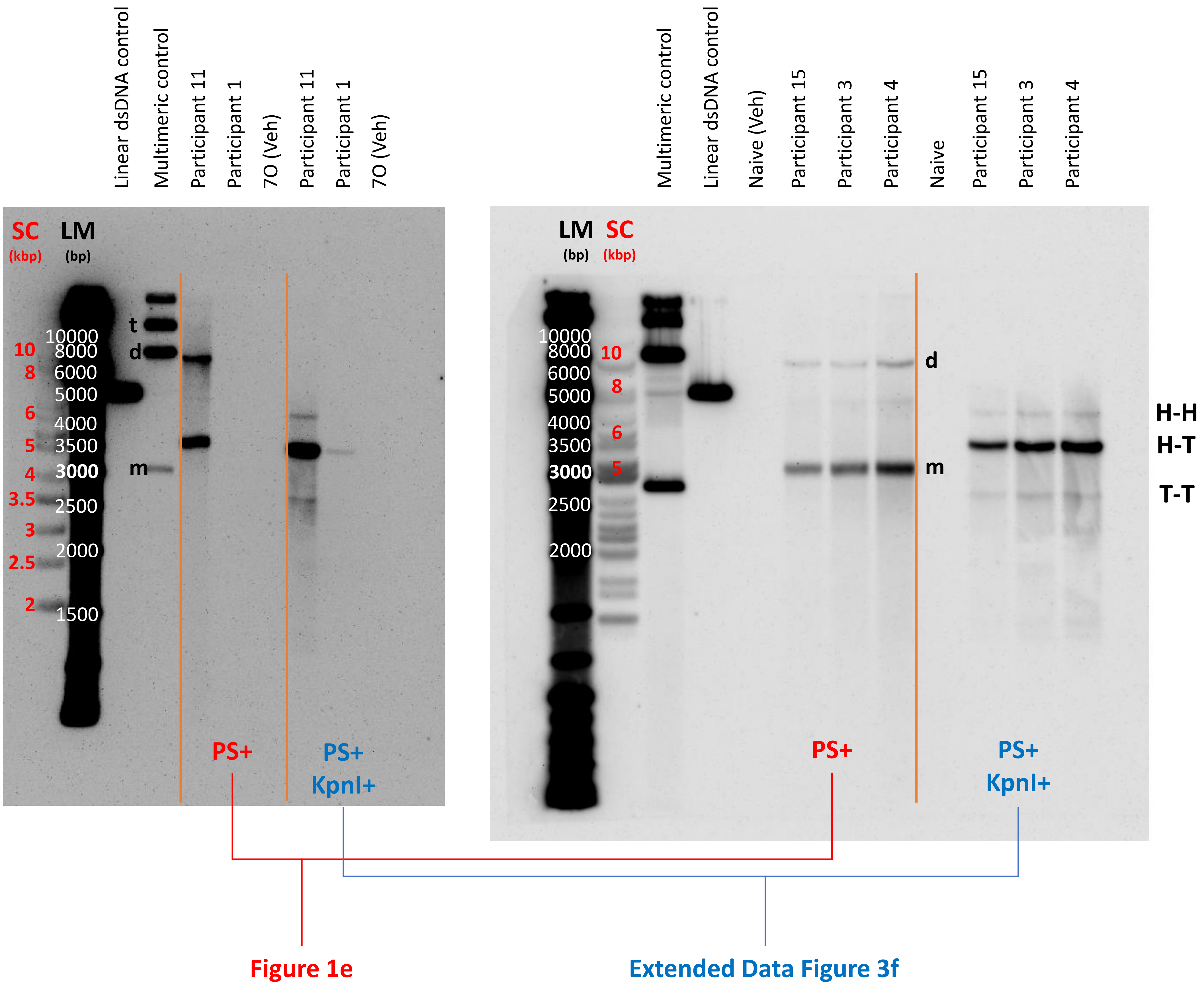

Supplement: Supplementary file 3 — Statistical source data (Figs. 1c,d). Unprocessed Southern blots (Fig. 1e). [file 41591_2022_1751_MOESM3_ESM.pdf]
