# Supplementary material for: Interindividual variability in transgene mRNA and protein production following adeno-associated virus gene therapy for hemophilia A
Source: Nat Med. 2022 Apr 11;28(4):789–97. doi: 10.1038/s41591-022-01751-0 (PMC9018415; doi:10.1038/s41591-022-01751-0)

Source data, Extended Data Figures 3a–3e

Quantification of circular vector genomes (DNA)

Circular multimeric episome quantification following PS-DNase or PS-DNase/KpnI digestion (mean copies/diploid genome)

		R2-R10 linked					
Participant	Week (biopsy)	no enzyme (lin + cir vg)	No enzyme +KpnI	with PS (circular vg, monomers + concatemers)	**Extended Data Fig 3a** with PS+KpnI (total circular vg, H-T only)	% Linked (overall)	% Linked (circular)
P1, 6e12	201	0.10	0.15	0.06	0.10	59.6%	68.3%
P11, 4e13	140	3.05	3.87	0.99	1.68	41.8%	44.2%
P15, 4e13	148	2.25	2.64	0.77	1.47	43.9%	54.8%
P3, 6e13	214	8.03	8.84	1.75	3.27	45.9%	52.2%
P4, 6e13	213	12.43	13.93	2.33	4.25	47.2%	51.9%

		R1-R11 linked					
				**Extended Data Fig 3e**			
Participant	Week (biopsy)	no enzyme (lin + cir vg)	No enzyme +KpnI	with PS (circular vg, monomers + concatemers)	**Extended Data Fig 3b** with PS+KpnI (total circular vg, H-T only)	% Linked (overall)	% Linked (circular)
P1, 6e12	201	0.09	0.16	0.05	0.10	71.0%	69.2%
P11, 4e13	140	2.71	3.52	0.94	1.66	43.2%	46.2%
P15, 4e13	148	2.01	2.61	0.73	1.29	46.3%	54.9%
P3, 6e13	214	7.19	8.72	1.65	3.11	47.2%	52.4%
P4, 6e13	213	11.08	13.47	2.05	4.24	47.4%	53.0%

		SQ			
Participant	Week (biopsy)	no enzyme (lin + cir vg)	No enzyme +KpnI	with PS (circular vg, monomers + concatemers)	**Extended Data Fig 3c** with PS+KpnI (total circular vg, H-T only)
P1, 6e12	201	0.14	0.33	0.09	0.19
P11, 4e13	140	7.07	15.97	2.75	5.94
P15, 4e13	148	4.69	10.26	1.68	4.28
P3, 6e13	214	15.39	35.27	4.24	10.99
P4, 6e13	213	24.02	52.05	5.23	14.03

		ITR fusion							
				**Extended Data Fig 3e**					
Participant	Week (biopsy)	no enzyme (lin + cir vg)	No enzyme +KpnI	with PS (circular vg, monomers + concatemers)	**Extended Data Fig 3d** with PS+KpnI (total circular vg, H-T only)	% Linked (overall)	% Linked (+PS-DNase)	% Linked +KpnI (overall)	% Linked +KpnI (+PS-DNase)
P1, 6e12	201	0.11	0.18	0.06	0.1	98.7%	93.2%	97.9%	97.0%
P11, 4e13	140	2.8	4.5	1.1	1.9	95.1%	95.1%	93.8%	95.2%
P15, 4e13	148	2.4	3.3	0.9	1.6	96.4%	97.0%	94.9%	97.3%
P3, 6e13	214	7.5	10.6	2.2	3.7	97.1%	96.4%	95.2%	95.7%
P4, 6e13	213	12.6	16.3	2.9	5.2	96.5%	96.2%	94.1%	96.5%

Source data, Extended Data Figure 3f

Southern blotting, uncropped images used in Fig. 1e and Extended Data Fig. 3f

bp, basepairs; d, dimer; H-H, head-to-head orientation; H-T, head-to-tail orientation; kbp, kilobase pairs; LM, linear markers; m, monomer; PS+, DNA samples treated with PS-DNase; PS+ KpnI+, DNA samples treated with PS-DNase followed by KpnI restriction enzyme digest; SC, supercoiled markers; T-T, tail-to-tail orientation

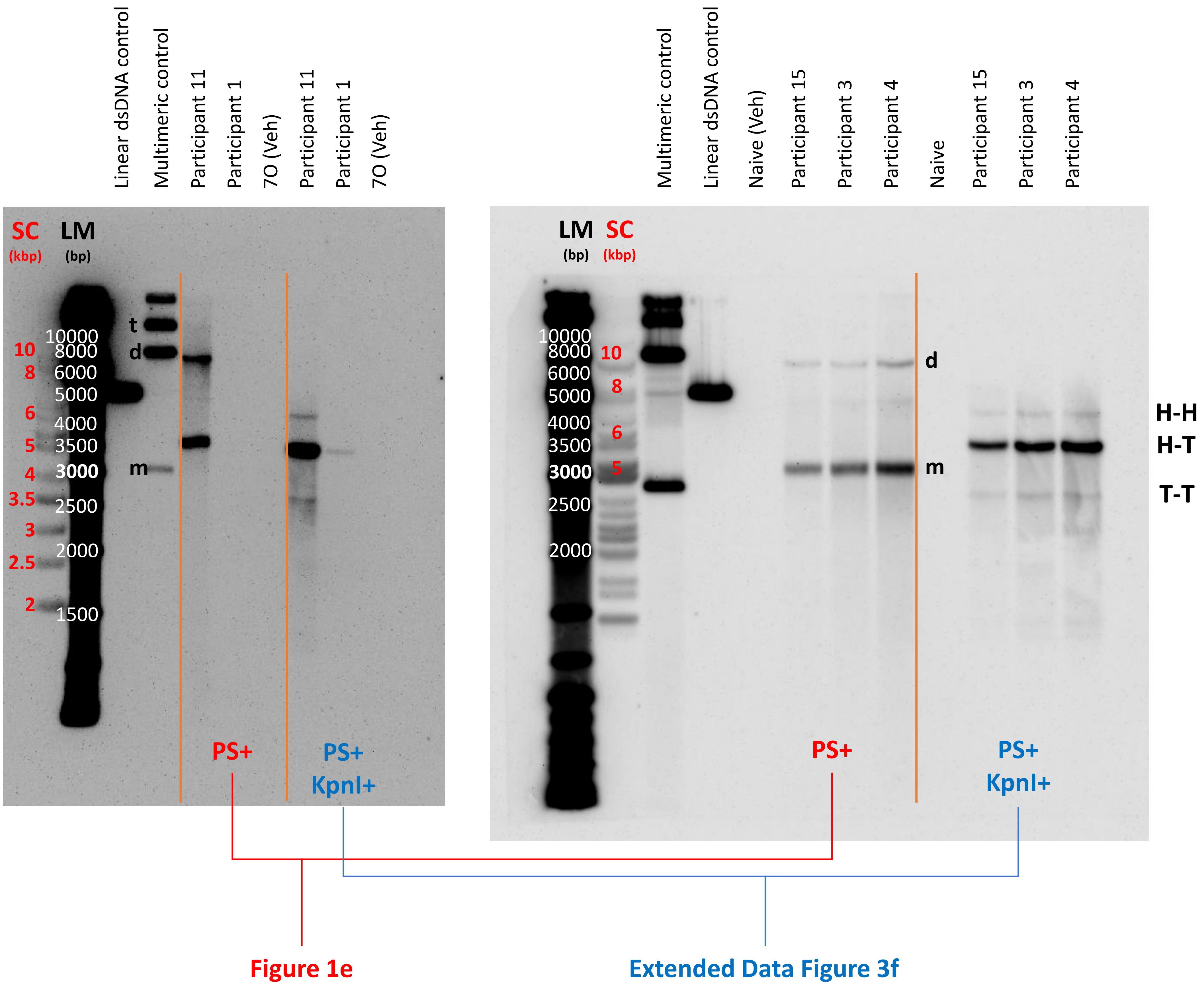

Supplement: Supplementary file 7 — Statistical source data (Extended Data Figs 3a–e). Unprocessed Southern blots (Extended Data Fig. 3f). [file 41591_2022_1751_MOESM7_ESM.pdf]
